# High temperature and cardiovascular disease in Australia under different climatic, demographic, and adaptive scenarios

**DOI:** 10.1093/eurheartj/ehaf117

**Published:** 2025-03-17

**Authors:** Jingwen Liu, Blesson M Varghese, Alana Hansen, Keith Dear, Timothy Driscoll, Ying Zhang, Geoffrey Morgan, Vanessa Prescott, Vergil Dolar, Michelle Gourley, Anthony Capon, Peng Bi

**Affiliations:** School of Public Health, The University of Adelaide, Level 4, 50 Rundle Mall Plaza, Rundle Mall, Adelaide, South Australia 5000, Australia; School of Public Health, The University of Adelaide, Level 4, 50 Rundle Mall Plaza, Rundle Mall, Adelaide, South Australia 5000, Australia; School of Public Health, The University of Adelaide, Level 4, 50 Rundle Mall Plaza, Rundle Mall, Adelaide, South Australia 5000, Australia; School of Public Health, The University of Adelaide, Level 4, 50 Rundle Mall Plaza, Rundle Mall, Adelaide, South Australia 5000, Australia; Sydney School of Public Health, The University of Sydney, Sydney, Australia; Sydney School of Public Health, The University of Sydney, Sydney, Australia; Sydney School of Public Health, The University of Sydney, Sydney, Australia; The University Centre for Rural Health, The University of Sydney, Sydney, Australia; Prevention and Environmental Health Unit, Australia Institute of Health and Welfare, Canberra, Australia; Burden of Disease and Mortality Unit, Australian Institute of Health and Welfare, Canberra, Australia; Burden of Disease and Mortality Unit, Australian Institute of Health and Welfare, Canberra, Australia; Monash Sustainable Development Institute, Monash University, Melbourne, Australia; School of Public Health, The University of Adelaide, Level 4, 50 Rundle Mall Plaza, Rundle Mall, Adelaide, South Australia 5000, Australia

**Keywords:** Climate change, Burden of diseases, High temperature, Cardiovascular diseases

## Abstract

**Background and Aims:**

Cardiovascular disease (CVD), the leading cause of death globally and in Australia, is sensitive to heat exposure. This study assesses the burden of CVD attributable to high temperatures across Australia and projects future burden in the context of climate change.

**Methods:**

Disability-adjusted life years for CVD, including years of life lost and years lived with disability, were sourced from the Australian Burden of Disease database. A meta-regression model was constructed using location-specific predictors and relative risks from prior literature to estimate relative risks of CVD mortality and morbidity due to high temperatures in the Australian context. The baseline CVD burden attributable to high temperatures in Australia for 2003–18 was calculated, and future burdens under two greenhouse gas emissions scenarios [Representative Concentration Pathways (RCP4.5 and RCP8.5)] for the 2030s and 2050s were projected, considering demographic changes and human adaptation.

**Results:**

During the baseline period, high temperatures accounted for 7.3% (95% confidence interval: 7.0%–7.6%) of the CVD burden in Australia, equivalent to 223.8 Disability-adjusted life years (95% confidence interval: 221.0–226.6) per 100 000 population. Future projections suggest a steady increase in the CVD burden across all scenarios examined. By the 2050s, under the RCP8.5 scenario that considers population growth and no adaptation, the total attributable burden of CVD is projected to more than double compared with the baseline, with the Northern Territory facing the most significant increase. These impacts could be mitigated with effective human adaptation to the warming climate.

**Conclusions:**

Higher temperatures are expected to exacerbate the burden of CVD. This study highlights the need for urgent adaptation and mitigation efforts to minimize the negative health impacts of a warming climate on CVD.

## Structured Graphical Abstract

**structured graphical abstract:**
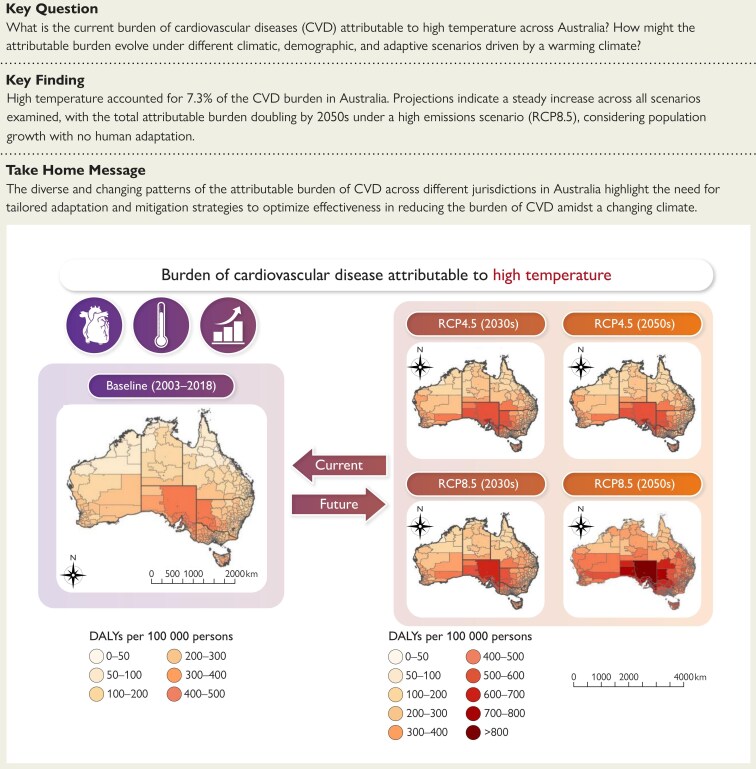
Comprehensive and comparative picture of the burden of cardiovascular diseases attributable to high-temperature exposure, contrasting baseline with future projections. The visual elements highlight the spatial and temporal trends and dynamics, demonstrating how the potential increase in the attributable burden of cardiovascular diseases in Australia is influenced by various climate scenarios, considering population growth with no human adaptation. CVD, cardiovascular disease; DALY, disability-adjusted life year; RCP, Representative Concentration Pathway.

## Introduction

A rise in anthropogenic greenhouse gases in the atmosphere has contributed to a detectable warming of the planet, with the global surface temperature in the last decade (2011–20) being 1.1°C higher than in 1850–1990.^1^ The Global Burden of Disease (GBD) Study 2019 reported that high temperature was responsible for 11.7 million disability-adjusted life years (DALYs) lost worldwide in 2019.^2^ High temperatures are more likely to pose a higher risk in susceptible populations such as people with chronic diseases [particularly cardiovascular diseases (CVDs)] and older people.^3–5^ As the leading cause of premature deaths globally, CVD accounted for an estimated 18.6 million lives lost in 2019.^6^ The resultant health impact is expected to increase significantly across the globe and in Australia, as shown by the recent increase in total CVD prevalence, deaths, illnesses, and healthcare expenditure.^6–9^ There is interest in projecting the impact of ambient temperatures on CVD due to the warming climate and an aging population to inform public health interventions and improve healthcare systems.^10–12^

Recent studies have evaluated health burdens associated with non-optimal temperatures^13,14^ and projected future health impacts (particularly mortality) attributable to high temperatures under different greenhouse gas emission (GHE) scenarios and population dynamics.^15,16^ However, the evidence on the future impact of high temperatures on CVD is still limited,^17,18^ and no study has projected the future burden of CVD incorporating years of life lost (YLL) and years lived with disability (YLD). Estimates of the variation in burden of disease attributable to high temperature are subject to uncertainty due to heterogeneity in population characteristics and health and climate conditions across regions.^13,19,20^ There is, therefore, a necessity to fill relevant knowledge gaps to develop and prioritize region-specific climate strategies.

Australia is among the world's most vulnerable nations to global warming, as evidenced by the increase in extreme heat events and average temperatures.^1^ Additionally, high-temperature-induced burden of CVD may amplify at an unprecedented rate in the future due to the longer life expectancy of an aging population.^8,21^ To our knowledge, no previous study has quantified the high-temperature-attributable burden of CVD across Australia, as measured in DALYs, including both YLL and YLD. This study estimates the high-temperature-attributable fatal and non-fatal burden of CVD in Australia by all jurisdictions for the period 2003–18 and projects the burden for the 2030s and 2050s, to obtain a national picture of the high-temperature-attributable burden of CVD in the context of climate change.

## Methods

The methodology we employed to estimate and project the burden of CVD attributable to high-temperature exposure is detailed in the Supplementary data, following our established methodological framework published previously.^22^ Our analysis was carried out using national data stratified into 2310 mutually exclusive spatial regions known as Statistical Areas Level 2 (SA2). We used the 2016 digital boundaries, which corresponded to the latest census data available during our defined baseline period (2003–18).^23^ Firstly, we estimated the annual average fatal and non-fatal burden of CVD for each of the SA2 areas from 2003 to 2018. Secondly, we fitted the exposure–response relationship for each location, assuming a log-linear monotonic increase in the relative risk (RR) for CVD per 1°C increase in high temperature above the theoretical minimum risk exposure distributions (TMREDs).^22^ We employed a meta-regression model,^12^ constructed using Stata (version 17.0).^24^ This model integrates pooled effect estimates (RRs) with varying lag structures sourced from studies elsewhere undertaken in the same climate zones as those in Australia.^13,14,22^ We incorporated location-specific meta-predictors (annual mean temperature, gross domestic product per capita, latitude, continent, and Köppen–Geiger climate zone), which have been shown to explain the heterogeneity of location-specific associations.^6,7^ Such model construction enables us to capture both short-term temperature effects and long-term exposure to increasing high temperature. The predictors were used to establish comparable settings between the location-specific RRs derived from the original studies^12–14^ and to estimate the SA2-specific RRs in Australia.^22^ Thirdly, we computed the high-temperature-related population attributable fractions (PAFs) by utilizing the location-specific RRs along with the corresponding observed and projected temperature data, as detailed in the Data sources section below. Assuming a constant exposure–response relationship for the projected periods,^25^ the calculations of PAFs were performed using previously developed code in Python.^22^ High-temperature exposure was defined as the days when the mean temperature exceeded the annual most frequent temperatures per SA2, representing the TMREDs.^26^ Finally, we calculated the high-temperature-attributable fatal and non-fatal burden of CVD in each location and compared annual average differences between the baseline (2003–18) and two future periods (2016–45 and 2036–65, hereafter referred to as ‘2030s’ and ‘2050s’).

We summarized and compared the changes across Australia, divided into six states [New South Wales (NSW), Queensland (Qld), Victoria (Vic), Western Australia, South Australia (SA), and Tasmania] and two territories (Australian Capital Territory and Northern Territory). This research evaluated two GHEs [Representative Concentration Pathways (RCP4.5 and RCP8.5)], while accounting for population ageing, and made assumptions about either a stable population size or population changes according to available projections from the Australian Bureau of Statistics (ABS).^27^ This approach allows us to examine the complex dynamics of future increase in the attributable burden of CVD, driven by a growing and aging population, ongoing warming climate, and the increasing frequency of high-temperature events. We also adjusted for different scenarios of human adaptation (none, partial, and full) by assuming different degrees of population acclimatization to increasing temperatures through the adjustment of TMREDs.^28^ The geographic information system ArcGIS Pro (version 3.1.0) was used to generate the graphical outputs for assessing the spatial distributions of the attributable burden of CVD.^29^

### Data sources

The annual Australian burden of CVD measured in DALYs, including both YLL and YLD, was sourced from the Australian Institute of Health and Welfare Burden of Disease database,^21^ using population-based records in four reference years (2003, 2011, 2015, and 2018). We used linear interpolation to fill the missing YLL and YLD values between years, based on the crude rate (per 100 000 population) per state and territory by age group (0–14, 15–44, 45–64, 65–74, and ≥75 years) per reference year.^30^

Daily data on mean temperature (*T*_mean_) was calculated from the high-resolution gridded observations (0.05° × 0.05°) of maximum and minimum temperature, obtained from the ‘Scientific and Information for Land Owners’ website.^31^ Future temperature projections were downloaded from the Commonwealth Scientific and Industrial Research Organisation database.^32^ We averaged the *T*_mean_ data from eight climate models for the RCP4.5 (a stabilization scenario with emissions peaking around 2040) and RCP8.5 (a scenario with continually rising emissions).^33^  Supplementary data online, *Table S1* summarizes details of the eight climate models. As recommended,^16,32^ we estimated the long-term trends in temperature and attributable fractions over 30-year periods.

We obtained the population data per SA2 from the ABS,^34^ including three projection series—Series A (increased migration, fertility, and life expectancy), Series B (current trends in migration, fertility, and life expectancy), and Series C (decreased migration, fertility, and life expectancy) (see Supplementary data online, *Figure S1*).^27^ We used Series B population projections for the main analysis and reported findings from Series A and C projections in the supplementary.^35^ We factored in the impact of an aging population on future burden CVD for each jurisdiction, based on the projected proportion of the population aged 65 or older in the population projections.

### Sensitivity analysis for uncertainty

Multiple sensitivity analyses were conducted to test uncertainty and robustness of the main findings. We addressed uncertainty regarding exposure–response curves, background burden of CVD, and high-temperature exposure projections by considering several factors in each analysis step. These factors included the use of linear and non-linear functions to predict RRs, changes in TMREDs, different climate projection models, different population trends, and adaptations. Further details and results are available in the Supplementary data.

## Results

### Descriptive data

Summary statistics of the annual average DALYs, YLLs, and YLDs for CVD in each state and territory during the baseline period are presented in Supplementary data online, *Table S2*. Measuring by DALYs, the states with the highest proportion of burden CVD were those with the largest populations—NSW and Vic, with 34.3% and 23.5% of the national DALYs, respectively. The Northern Territory (NT) and Qld had the highest age-standardized rate of 5224.2 DALYs per 100 000 population and 3059.4 DALYs per 100 000 population, respectively.

**Figure 1:**
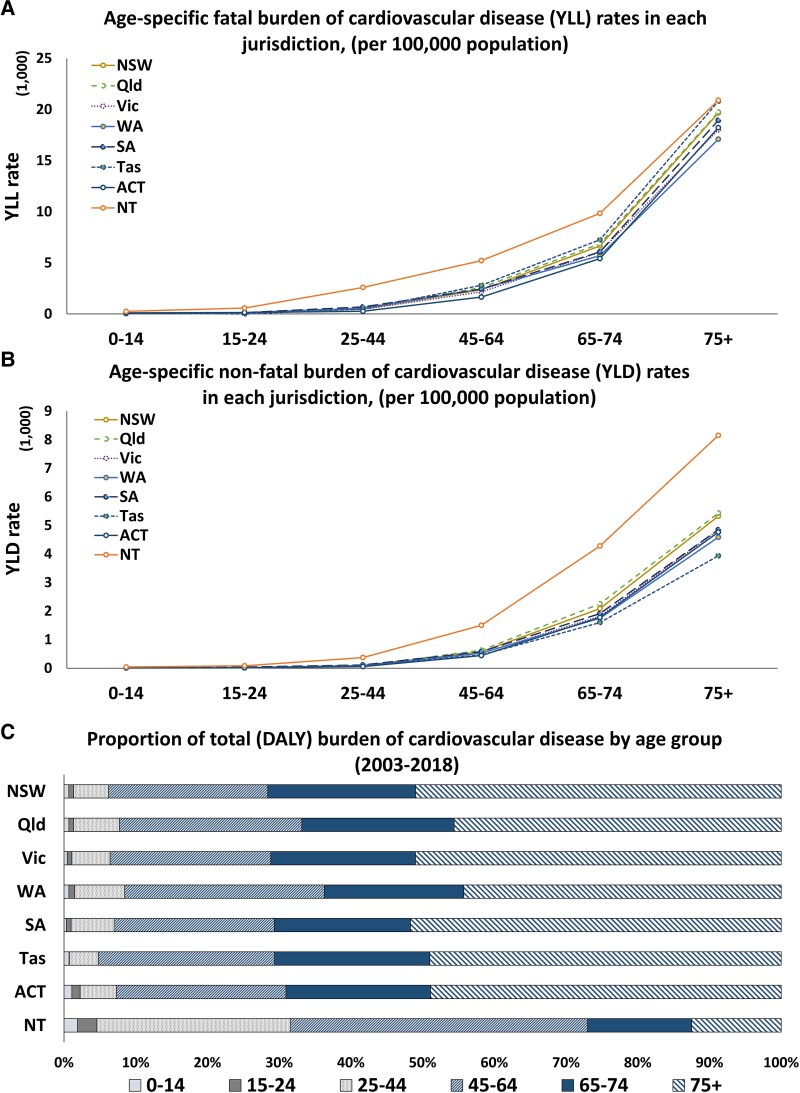
Comparison of fatal and non-fatal burden of cardiovascular disease by age groups and jurisdictions. This figure provides a detailed comparison of the burden of cardiovascular diseases across different age groups (0–14, 15–24, 25–44, 45–64, 65–74, and over 75) and jurisdictions. It includes both fatal cardiovascular disease rates (years of life lost) and non-fatal cardiovascular disease rates (years lived with disability), highlighting trends and disparities among age groups in each Australian state and territory (*A* and *B*). The figure also includes graphical elements to show the relative proportion of the total cardiovascular disease burden represented by each age group (*C*). ACT, Australian Capital Territory; CVD, cardiovascular disease; DALY, disability-adjusted life year; NSW, New South Wales; NT, North Territory; Qld, Queensland; SA, South Australia; Tas, Tasmania; Vic, Victoria; WA, Western Australia; YLD, years lived with disability; YLL, years of life lost

*Figure 1* presents the rates (per 100 000 population) and proportion (%) for fatal and non-fatal burden CVD by age group. As expected, the rates of burden of CVD increase with age, as shown in *Figure 1A* and *B*. Notably, rates were higher in the NT compared with other jurisdictions, with these gaps increasing for both the fatal and non-fatal burdens of CVD from age 15 onwards. Additionally, among all jurisdictions, only the NT had a higher proportion (73%) of total burden CVD (fatal and non-fatal) in the younger age group (0–64 years) than the older population (27%) as depicted in *Figure 1C*. Supplementary data online, *Figure S2* shows the DALY rates by age group.

### Estimated high-temperature-attributable burden of cardiovascular disease during baseline

**Figure 2:**
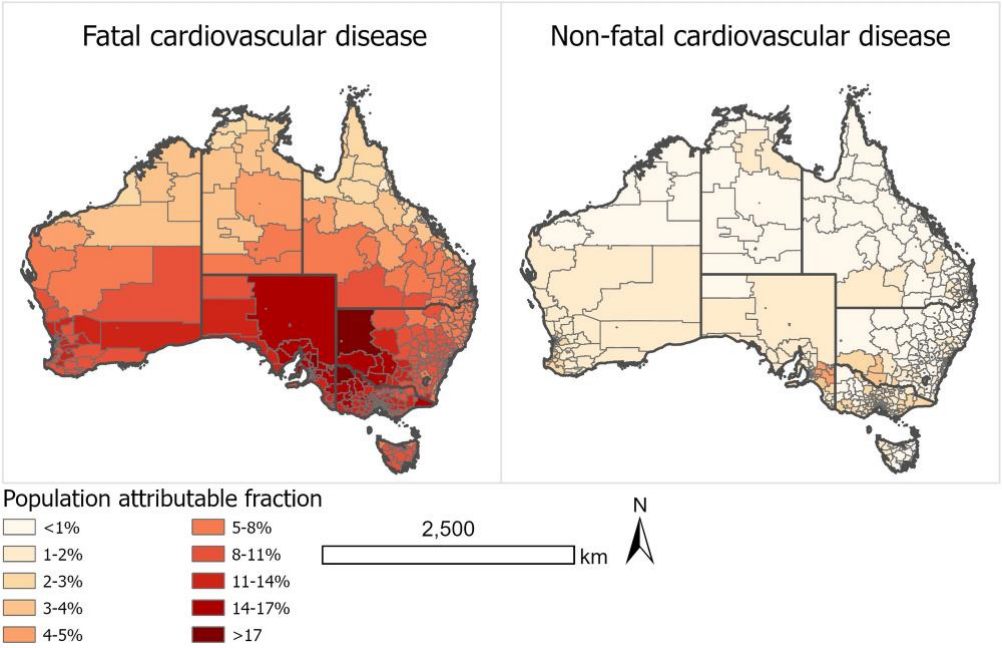
Population attributable fraction for both fatal and non-fatal cardiovascular diseases due to high-temperature exposure during the baseline. This figure shows the population attributable fraction for both fatal and non-fatal burdens of cardiovascular diseases due to high-temperature exposure during the baseline period, categorized by the Statistical Areas Level 2 within each state and territory in Australia. Data are averaged across the baseline period from 2003 to 2018, providing spatial distribution of the impact of high-temperature on the burden of cardiovascular disease across Australia

The geographic distribution of RR per unit increase in high temperature at SA2 level for CVD mortality and morbidity is presented in Supplementary data online, *Figure S3*. It is observed that regions in Southern Australia, although generally cooler, are associated with higher RRs, especially for CVD mortality. Supplementary data online, *Figure S4* illustrates the distribution of threshold temperatures (TMREDs) for each SA2 across Australia, ranging from 7.8°C in the south to 31.7°C in the north. The PAFs (*Figure 2*) demonstrate the percentage reduction in the burden of CVD that would occur for a population if high-temperature exposure is reduced to its theoretical minimum.^21^ The PAF estimations are affected by the RRs used, as well as the prevalence of high-temperature exposure at the location, i.e. the number of days in a year exceeding the TMREDs.

*Table 1* shows that during the baseline period (2003–18), the average annual rate of the burden of CVD attributable to high temperatures in Australia was 223.8 DALYs per 100 000 population, with 7.3% attributable to high temperatures. There was an annual average of 49 483 years of healthy life lost, with the fatal proportion being 97% and the non-fatal 3%. States with the largest populations and burden of CVD, namely Vic and NSW, had the greatest number of high-temperature-attributable DALYs (15 621 and 14,001, respectively). The lowest was in the NT (214 DALYs). South Australia had the highest rate of high-temperature-attributable burden of CVD at 416.6 DALYs per 100 000 population and the highest proportion of burden of CVD attributable to high temperature (11.9% of the observed burden) (*Table 1*). Supplementary data online, *Figure S5* depicts the regional differences in the rate (per 100 000 population) of high-temperature-attributable burden of CVDs in Australia.

**Table 1:** Annual mean temperature (range) and burden of cardiovascular diseases attributable to high temperatures, by state and territory in Australia, 2003–18

State/territory	*T*_mean_ (range)	Attributable YLLs (SE)	Attributable YLDs (SE)	Attributable DALYs (SE)	% of attributable DALYs (SE)	Attributable DALYs rate, per 100 000 persons^a^ (SE)
NSW	17.6 (6.5–33.2)	13 553.6 (613.8)	447.2 (24.6)	14 000.8 (634.8)	6.03 (0.30)	194.8 (9.2)
Qld	21.5 (10.0–33.5)	4938.5 (276.9)	166.9 (12.0)	5105.4 (286.6)	3.76 (0.20)	117.0 (7.7)
Vic	15.0 (4.4–34.5)	15 122.6 (511.8)	498.8 (23.6)	15 621.4 (532.6)	9.77 (0.35)	281.9 (9.6)
WA	18.9 (7.8–34.3)	5748.2 (199.9)	125.3 (4.9)	5873.5 (203.7)	9.79 (0.37)	257.0 (10.0)
SA	16.7 (6.0–36.8)	6584.4 (189.2)	184.1 (6.3)	6768.5 (193.2)	11.88 (0.35)	416.6 (13.6)
Tas	12.1 (2.3–28.9)	1301.0 (48.7)	25.4 (1.3)	1326.5 (49.8)	7.28 (0.28)	262.6 (9.3)
ACT	13.8 (0.6–30.4)	551.4 (31.6)	25.3 (1.4)	576.7 (32.9)	7.46 (0.38)	157.7 (9.1)
NT	26.8 (15.7–33.6)	205.3 (9.8)	8.7 (0.6)	214.0 (10.3)	2.58 (0.13)	94.5 (4.8)
Australia	17.9 (6.7–33.6)	48 001.5 (1007.8)	1481.7 (59.6)	49 483.2 (1052.9)	7.29 (0.19)	223.8 (5.7)

ACT, Australian Capital Territory; NSW, New South Wales; NT, North Territory; Qld, Queensland; SA, South Australia; Tas, Tasmania; Vic, Victoria; WA, Western Australia.

^a^Averaged total across baseline period and standard deviation (SD).

### Estimated projection of future high-temperature-attributable burden of cardiovascular disease

Supplementary data online, *Table S3* presents the projected mean temperatures for each state and territory under the two GHE scenarios (RCP4.5 and RCP8.5). The mean temperature is expected to increase over time across Australia, with a steeper gradient under RCP8.5 than RCP4.5. A graphical representation of the temperature trend is shown in Supplementary data online, *Figure S6*. According to population age structure data under the ABS Series B projection (current trends in migration, fertility, and life expectancy), the proportion of the population aged ≥65 years across jurisdictions would rise from 7.5%–19.7% in 2018 to 10.9%–26.4% by 2060, indicating an ageing population (see Supplementary data online, *Table S4*). The projected trend by age groups in each jurisdiction is displayed in Supplementary data online, *Figure S7*.^27^

**Figure 3:**
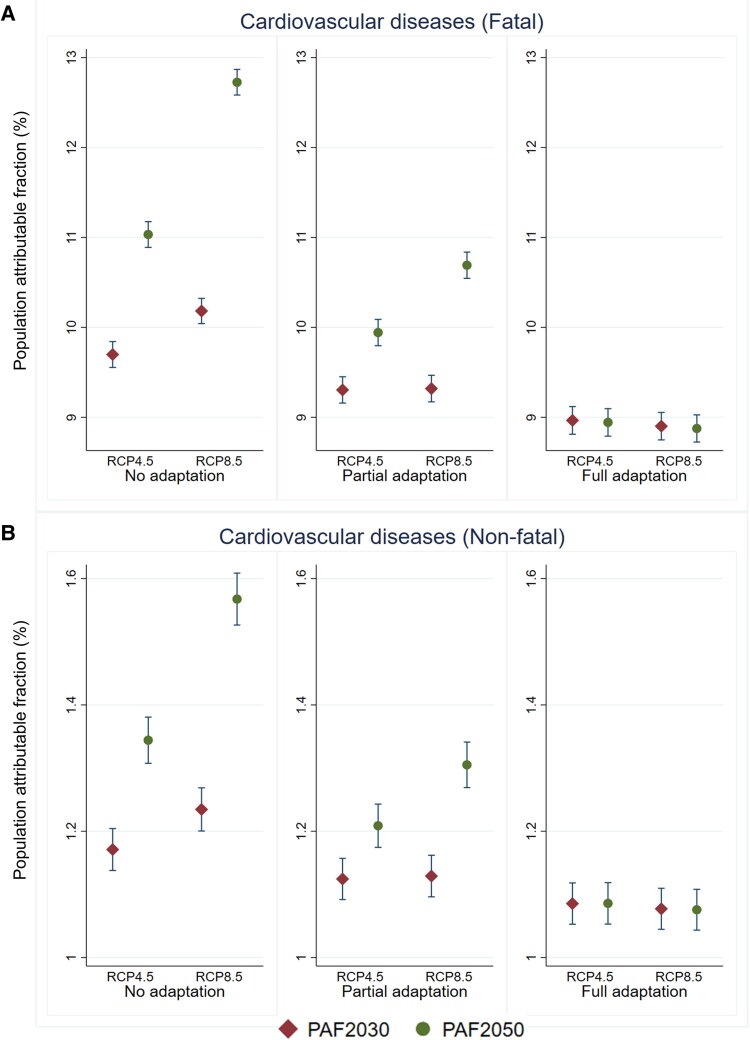
Projected population attributable fraction for both fatal and non-fatal cardiovascular diseases due to increasing high-temperature exposure in Australia in future periods. This figure shows the comparison of the population attributable fraction for fatal (*A*) and non-fatal (*B*) cardiovascular disease due to high-temperature exposure in Australia, centred on future periods of 2030s and 2050s. It shows mean values and 95% confidence intervals under two Representative Concentration Pathways (RCP4.5 and RCP8.5), along with scenarios of human adaptation to climate change (none, partial, and full). PAF, population attributable fraction; RCP, Representative Concentration Pathway

*Figure 3* shows the mean of projected PAFs for high-temperature-attributable fatal and non-fatal burden of CVD in 2030s and 2050s, for three adaptation scenarios (none, partial, and full) under RCP4.5 and RCP8.5. Across all scenarios examined, we observed higher PAFs for fatal than non-fatal CVD, with the differences ranging from 7.7% to 11.6%. We observed decreasing trend in the PAFs as adaptation increased from 0% (no adaptation) to 100% (full adaptation). For the fatal burden of CVD, the PAFs ranged 9.7%–12.7% when there was no adaptation, 9.3%–10.7% under partial adaptation, and 8.9%–9.0% under full adaptation (*Figure 3A*). Similarly, the PAFs for non-fatal burden of CVD ranged 1.2%–1.6% assuming no adaptation, 1.1%–1.3% under partial adaptation, and 1.1% under full adaptation (*Figure 3B*). Supplementary data online, *Figure S8* displays the variations in the estimated PAFs for each state and territory.

*Table 2* compares the results of three adaptation scenarios for both RCP4.5 and RCP8.5 and two population scenarios, with the projected increase in the proportion of burden of CVD linked to high temperatures for 2030s and 2050s compared with baseline, for each jurisdiction.

**Table 2:** Projected high-temperature-attributable burden of cardiovascular disease for future periods centred on 2030s and 2050s (annual averaged number of disability-adjusted life years) and the projected percentage change (%) compared with the baseline (2003–18), under scenarios of constant population and medium population growth, adaptation scenarios (none, partial, and full), and two Representative Concentration Pathways (RCP4.5 and RCP8.5)

RCPs	State/territory	Projected burden of cardiovascular disease attributable to high temperature, DALYs (%)											
		Constant population						Medium population growth (Series B projection)					
		No adaptation		Partial adaptation		Full adaptation		No adaptation		Partial adaptation		Full adaptation	
		2030s	2050s	2030s	2050s	2030s	2050s	2030s	2050s	2030s	2050s	2030s	2050s
4.5	NSW	15 745.0 (12.5%)	18 365.5 (31.2%)	15 631.9 (11.6%)	16 791.6 (19.9%)	15 590.7 (11.4%)	15 267.2 (9.0%)	25 272.5 (80.5%)	39 051.5 (178.9%)	25 122.7 (79.4%)	35 763.7 (155.4%)	25 075.5 (79.1%)	32 531.2 (132.4%)
	Qld	6497.1 (27.3%)	8004.9 (56.8%)	5612.7 (9.9%)	6355.6 (24.5%)	4831.1 (−5.4%)	4878.1 (−4.5%)	11 791.7 (131.0%)	19 948.1 (290.7%)	10 183.4 (99.5%)	15 857.4 (210.6%)	8767.4 (71.7%)	12 169.9 (138.4%)
	Vic	16 208.4 (3.8%)	17 948.7 (14.9%)	16 236.6 (3.9%)	17 163.1 (9.9%)	16 303.6 (4.4%)	16 358.9 (4.7%)	27 944.0 (78.9%)	43 825.9 (180.6%)	27 997.6 (79.2%)	41 953.0 (168.6%)	28 141.7 (80.1%)	40 005.8 (156.1%)
	WA	6436.1 (9.6%)	7045.0 (19.9%)	5943.0 (1.2%)	6182.6 (5.3%)	5505.6 (−6.3%)	5373.8 (−8.5%)	11 453.0 (95.0%)	17 814.0 (203.3%)	10 552.1 (79.7%)	15 608.9 (165.7%)	9757.0 (66.1%)	13 561.6 (130.9%)
	SA	7171.0 (5.9%)	7704.6 (13.8%)	7088.7 (4.7%)	7354.3 (8.7%)	7009.3 (3.6%)	7017.7 (3.7%)	10 497.3 (55.1%)	13 511.4 (99.6%)	10 381.3 (53.4%)	12 905.2 (90.7%)	10 273.4 (51.8%)	12 317.9 (82.0%)
	Tas	1391.0 (4.9%)	1637.6 (23.5%)	1292.4 (−2.6%)	1412.3 (6.5%)	1218.2 (−8.2%)	1240.0 (−6.5%)	2112.4 (59.2%)	2845.1 (114.5%)	1963.3 (48.0%)	2454.7 (85.1%)	1852.2 (39.6%)	2155.2 (62.5%)
	ACT	609.5 (5.7%)	687.6 (19.2%)	614.7 (6.6%)	658.6 (14.2%)	610.5 (5.8%)	635.8 (10.2%)	1177.9 (104.3%)	1884.8 (226.8%)	1184.5 (105.4%)	1804.5 (212.9%)	1172.8 (103.4%)	1738.5 (201.5%)
	NT	348.0 (65.3%)	457.3 (117.2%)	287.0 (36.3%)	327.9 (55.7%)	228.5 (8.6%)	233.0 (10.7)	531.1 (152.3%)	948.0 (350.3%)	434.9 (106.6%)	670.5 (218.5%)	342.3 (62.6%)	470.2 (123.3%)
	National	54 406.1 (9.9%)	61 851.2 (25.0%)	52 707.1 (6.5%)	56 246.2 (13.7%)	51 297.1 (3.7%)	51 004.6 (3.1%)	90 779.7 (83.5%)	139 828.9 (182.6%)	87 819.8 (77.5%)	127 017.9 (156.7%)	85 382.3 (72.5%)	114 950.4 (132.3%)
8.5	NSW	16 695.4 (19.2%)	21 918.1 (56.5%)	15 985.2 (14.2%)	18 370.4 (31.2%)	15 429.7 (10.2%)	15 147.1 (8.2%)	26 793.8 (91.4%)	46 603.8 (232.9%)	25 695.7 (83.5%)	39 102.2 (179.3%)	24 816.2 (77.2%)	32 296.6 (130.7%)
	Qld	7283.2 (42.7%)	9948.0 (94.9%)	5550.3 (8.7%)	7236.1 (41.7%)	4802.4 (−5.9%)	4872.8 (−4.6%)	13 200.7 (158.6%)	24 758.3 (384.9%)	10 066.3 (97.2%)	18 027.8 (253.1%)	8722.3 (70.8%)	12 153.9 (138.1%)
	Vic	16 757.3 (7.3%)	19 969.8 (27.8%)	16 554.9 (6.0%)	17 991.1 (15.2%)	16 255.6 (4.1%)	16 098.4 (3.1%)	28 894.2 (85.0%)	48 747.0 (212.1%)	28 577.7 (82.9%)	43 972.0 (181.5%)	28 077.9 (79.7%)	39 379.3 (152.1%)
	WA	6522.7 (11.1%)	7784.0 (32.5%)	5690.5 (−3.1%)	6533.0 (11.2%)	5390.7 (−8.2%)	5406.5 (−8.0%)	11 613.8 (97.7%)	19 649.2 (234.5%)	10 103.6 (72.0%)	16 483.1 (180.6%)	9562.9 (62.8%)	13 628.5 (132.0%)
	SA	7371.7 (8.9%)	8436.3 (24.6%)	7128.3 (5.3%)	7701.2 (13.8%)	6986.2 (3.2%)	7025.0 (3.8%)	10 786.7 (59.4%)	14 789.5 (118.5%)	10 441.6 (54.3%)	13 512.2 (99.6%)	10 233.4 (51.2%)	12 330.7 (82.2%)
	Tas	1475.8 (11.3%)	1806.4 (36.2%)	1295.0 (−2.4%)	1490.1 (12.3%)	1215.1 (−8.4%)	1242.8 (−6.3%)	2242.6 (112.7%)	3138.5 (136.6%)	1970.7 (48.6%)	2590.0 (95.3%)	1849.8 (39.5%)	2160.5 (62.9%)
	ACT	634.7 (10.1%)	802.6 (39.2%)	620.3 (7.6%)	706.6 (22.5%)	606.5 (5.2%)	608.3 (5.5%)	1226.7 (112.7%)	2200.8 (281.6%)	1195.0 (107.2%)	1933.8 (235.3%)	1166.9 (102.4%)	1671.5 (189.9%)
	NT	381.8 (81.4%)	583.0 (176.9%)	255.8 (21.5%)	385.0 (82.9%)	218.8 (3.9%)	234.5 (11.4)	584.6 (177.7%)	1208.0 (473.8%)	385.4 (83.0%)	787.1 (273.9%)	327.1 (55.4%)	472.1 (124.2%)
	**National**	57 122.6 (15.4%)	71 248.3 (44.0%)	53 080.3 (7.3%)	60 413.4 (22.1%)	50 905.1 (2.9%)	50 635.3 (2.3%)	95 343.0 (92.7%)	161 095.1 (225.6%)	88 436.0 (78.7%)	136 408.3 (175.7%)	84 756.3 (71.3%)	114 093.1 (130.6%)

Estimates are the mean across eight climate models.

### Climate change

Given the higher increase in mean temperature under RCP8.5 compared with RCP4.5 over time, the high-temperature-attributable burden of CVD was projected to be generally higher under RCP8.5. For example, without any human adaptation or change in population, the burden of CVD attributable to high temperature is projected to be 5.0% higher in the 2030s (54 406.1 DALYs for RCP4.5 vs. 57 122.6 DALYs for RCP8.5) and 15.2% higher in the 2050s (61 851.2 DALYs for RCP4.5 vs. 71 248.3 DALYs for RCP8.5) higher in 2050s. However, in some jurisdictions, a decrease in high-temperature-attributable DALYs due to CVD was observed under the scenario of constant population and 100% human adaptation (*Table 2*).

### Changes in population

When comparing scenarios of constant population with those assuming changes in future population (population growth and aging), the results suggest that changes in future demographics will make a substantial contribution to the increase in the high-temperature-attributable burden of CVD. It is estimated that the percentage increase in the total burden of CVD attributable to high temperatures will rise from 15.4% to 92.7% by 2030s and from 44.0% to 225.6% by 2050s when no human adaptation is assumed (*Table 2*). To provide further context for the results, the contribution of changes in future demographics (population growth and aging) to the excess future high-temperature-attributable burden of CVD in relation to the baseline was calculated (see Supplementary data online, *Table S5*). The contribution of changes in population size and age structure accounted for over 80% of the projected increase in the burden of CVD associated with high-temperature exposure across all climate change scenarios examined.

### Human adaptation

The assumption of human adaptation has resulted in important differences in projections of the level of exposure to high temperature in the population. For instance, under the scenario of high GHEs (RCP8.5), the projected percentage increase in the high-temperature-attributable burden of CVD shifted from 225.6% (534.9 DALYs per 100 000 population) with no human adaptation to 175.7% (452.7 DALYs per 100 000 population) with partial (50%) human adaptation and further decreased to 130.6% (378.4 DALYs per 100 000 population) with full (100%) human adaptation, when accounting for changes in future demographics by 2050s (*Table 2*).

### Regional variation

In addition, we observed important differences across jurisdictions and scenarios. While SA is projected to continue having the highest rate and proportion of high-temperature-attributable burden of CVD by the 2030s, NT is expected to experience the highest percentage increase, particularly if no human adaptation is assumed (*Table 2*). Accordingly, by the 2050s, NT is projected to have the highest rate and proportion of burden of CVD attributable to high-temperature exposure in Australia, dependent on the extent of human adaptation. In contrast, SA shows little difference regardless of human adaptations. A visual representation of the geographic changes in burden of CVD rates due to future high-temperature exposure is provided in Supplementary data online, *Figure S8*. Supplementary data online, *Tables S6* and *S7* demonstrate differences in the DALY rate (per 100 000 population) and proportion of burden of CVD that can be attributed to high temperatures between jurisdictions under different scenarios.

### Sensitivity analyses

Sensitivity analyses using alternative TMREDs, different functional forms of exposure–response associations (including quadratic and cubic polynomial regression for non-linear relationships), and alternative RRs adjusted for various predictors demonstrate that the outputs of our findings (PAFs and attributable burden of CVD) remained fairly stable and consistent, regardless of the modelling choices during the baseline period (see Supplementary data online, *Table S8*). Additionally, the sensitivity analyses for future periods, factoring in different climate models and population projections, further support the robustness of our findings, as detailed in Supplementary data online, *Table S9*.

## Discussion

This study presents a novel contribution to the field by evaluating the observed and projected burden of CVD attributable to high temperature in Australia. The assessment includes results from over 2000 geographical areas in Australia covering all jurisdictions and generates a comprehensive national picture of the burden of CVD under different climate, demographic, and adaptation scenarios (*Structured Graphical Abstract*). To our best knowledge, this is the first such study globally.

Results of this investigation show that during the baseline period (2003–18), the observed burden of CVD in Australia that can be attributed to high temperature is 7.3%, which is dominated by the fatal burden. Notably, we observed spatial heterogeneity in which the southern regions (SA and Vic), characterized by higher RRs and less adaptation to high-temperature exposures (lower TMREDs), exhibited a higher attributable burden of CVD. The patterns are expected to persist into the future and could potentially be linked to demographic vulnerabilities, such as an older population,^12^ or to infrastructural factors like urban heat islands, which intensify the effects of high temperatures.^36^ Our projections compared scenarios of constant population with those with changes in population size and age structure, indicating that the burden of CVD attributed to high-temperature exposure would substantially increase due to population growth and ageing, particularly under the high GHEs scenario (RCP8.5). Assuming no human adaptation and keeping all other factors constant, the proportion of burden of CVD attributed to high-temperature exposure is projected to increase to 9.1% and 10.5% by 2050s under RCP4.5 and RCP8.5, respectively (see Supplementary data online, *Table S6*). However, as the extent of population adaptation increases from no to full (100%) adaptation, a steady decrease in PAFs is observed. These effects are more pronounced in the NT, which has a warm to hot climate^19^ and a high proportion of the population with socioeconomic and health challenges,^21^ suggesting both climate change mitigation strategies and human adaptation actions are urgently needed.

Previous studies on the burden of disease associated with high-temperature exposure have primarily focused on fatal health burdens (YLLs).^13,14,37,38^ In contrast, the present study has examined both fatal (YLLs) and non-fatal (YLDs) burdens using Australian-specific data for a comprehensive picture.^21,39^ Our estimated proportion of the total high-temperature-attributable YLLs for CVD is somewhat higher than the GBD estimate for Australia during the baseline period, with an annual average of 7.1% and 0.94%, respectively.^13^ However, differences in data sources, modelling approaches, and reference temperatures (TMRED) used to determine exposure^19^ make comparisons difficult. For instance, while the GBD study focused on same-day temperature effects,^13^ our analyses have expanded to include lagged temperature effects for temperature–CVD associations. By pooling location-specific RRs from prior studies that utilized distributed lag non-linear models, we can more effectively capture the complex dynamics of temperature fluctuations with varying lag sturctures.^12^ This consideration is particularly important for understanding the effects of temperature on CVD, as it accounts for potential mortality displacement or ‘harvesting’ when assessing heat-related deaths.^40^ Additionally, relying on data from cities in other countries as proxies can also contribute to differences in results.^13,14,39^ Nevertheless, our study offers important insights through a transferable methodology for quantifying the burden of CVD attributable to high temperature. This approach can be extended to other diseases and settings where public data are available,^22^ providing essential evidence that necessitates enhanced public health preparedness and response in the context of a warming climate.

Our projections align with previous studies that suggest that the health burden related to high temperature will increase in coming decades, with hotter regions facing more substantial impacts (steeper increase in PAFs),^15,41^ particularly under RCP8.5. Our findings indicate that together with the extent of adaptation to the warming climate, changes in population size and aging structure will likely be critical factors in determining the future excess burden of CVD caused by high temperatures.^41^ Population ageing is reportedly the primary factor contributing to the proportions of people susceptible to CVD,^25^ and studies have found that adaptation alone would not be sufficient to offset the increased health risks associated with warming temperatures, particularly under RCP8.5.^42^ Accordingly, it is important for policymakers and service providers to work collaboratively with the community to address vulnerability in the aging population. Health promotion efforts to increase fitness and reduce chronic disease risks in the elderly may help minimize heat-induced cardiovascular health risks in this demographic.^43,44^

In the present study, the comparisons between scenarios highlighted the important role of strategies for adapting to a warming climate. As adaptation capacity can be related to socioeconomic status, financial support for cooling and subsidies for air conditioning/electricity costs may likely benefit those with fewest resources.^12,43^ Furthermore, as patients with CVD are more at risk during hot weather, it is prudent for clinicians to provide practical advice relating to increasing fluid intake and plant-based diets, reducing outdoor activity levels, and guidelines for storing heat-sensitive medications.^43,45^ With the RCP8.5 scenario assuming high population growth, higher GHEs, and high energy consumption without climate change policies,^33^ the challenges ahead could also be mitigated with multidisciplinary industry and transportation collaborations linking renewable and climate-friendly energy resources to reduce anthropogenic GHEs.^46^ Furthermore, climate models predict very different levels of warming post-2050s, depending on the actions we take in the near future. This highlights the necessity for immediate and coordinated efforts in mitigation.^47^

Some limitations should be acknowledged. Our method of estimating RRs has limitations, as with previous studies.^13,14^ The RRs were sourced from international literature and location-specific meta-predictors in Australia.^22^ While the predictors explain most of the heterogeneity in the temperature–health association between regions,^14^ other potential effect modifiers may have been unaccounted for.^21,48^ It is also worth noting that heterogeneity in climate models and regional variations in temperature distributions and population vulnerability (such as socioeconomic status and healthcare access) may contribute to uncertainty in estimates.^28,41,44^ Moreover, this study assumes that human adaptation corresponds with increases in the magnitude of TMREDs, which may oversimplify the inherent complexities of adaptation, as it involves a myriad of socioeconomic, cultural, and infrastructure factors that vary across communities.^41,42^ Our projections, derived from the best available data from the ABS,^27^ may not account for potentially unexpected demographic shifts, such as the impacts on overseas migration in Australia observed with COVID-19.^49^ Additionally, even in the post-COVID era, the long-lasting effects of the pandemic on key CVD health behaviours (diet quality, tobacco use, and mental health),^50^ which are related to cardiometabolic disorders and subsequently the burden of CVD, remain uncertain.^51^ Therefore, future studies are warranted to more comprehensively assess the dynamic and complex aspects of human adaptation to climate change considering disparities in vulnerability and inequity across different Australian regions and communities.

In conclusion, this study finds that the impact of high temperatures on the burden of CVD is expected to increase considerably compared with the baseline, especially in the absence of human adaptation. As CVD is the leading cause of premature death globally,^6^ our result highlights the urgent need for implementing effective heat adaptation and mitigation strategies in public health policy. The findings are crucial for directing targeted public health planning efforts aimed at enhancing the adaptive capacities of older communities, increasing awareness to reduce the health burden of CVD,^44^ and reducing GHEs to prevent the potential loss of healthy life years. Future studies are needed to comprehensively assess the complex aspects of incorporating measures of adaptation and attenuation effects^41,52^ and further investigate the observed heterogeneity between regions.

## Supplementary Material

ehaf117_Supplementary_Data

## Declarations

### Data Availability

No data were generated or analysed for or in support of this paper.

### Pre-registered Clinical Trial Number

None supplied.
