# Supplementary material for: High temperature and cardiovascular disease in Australia under different climatic, demographic, and adaptive scenarios
Source: Eur Heart J. 2025 Mar 17;46(19):1852–62. doi: 10.1093/eurheartj/ehaf117 (PMC12075934; doi:10.1093/eurheartj/ehaf117)
Supplement: ehaf117_Supplementary_Data [file ehaf117_supplementary_data.zip › European Heart Journal_mmc1_R2.docx]

**Supplementary appendix**

Supplement to: High temperature and cardiovascular disease in Australia under different climatic, demographic and adaptive scenarios

**Data Analysis Methods**

**Stage 1: estimation of the burden of cardiovascular disease (CVD)**

We applied a downscaling procedure to quantify the burden of CVD at the SA2 level. Firstly, we determined the proportion of the population in each SA2 that corresponds to its respective state/territory or national population during the baseline period (2003-2018). Next, we multiplied this proportion by burden of CVD of the corresponding state/territory or national level, resulting in the YLLs and YLDs for each SA2. This downscaling approach assumes that the DALYs of SA2 areas in one jurisdiction would maintain the same proportion of population, which has been used in related researches.^1,2^

**Stage 2: estimation of the exposure-response relationships and thresholds**

Since there were no RRs covering all Australian geographical zones available locally, we estimated the association between high temperatures and CVD using data obtained from our systematic review of previous international studies.^3,4^ For a comparable analysis, we included studies that used daily Tmean as their exposure indicator only, and standardised the RRs associated with 1°C increase in mean temperature. Additionally, we collected the location-specific meta-predictors, such as annual mean temperature, GDP per capita, latitude, continent, and Köppen-Geiger climate zone.^5^ These meta-predictors have been shown to explain the heterogeneity of location-specific associations.^6,7^ Subsequently, we constructed a meta-regression model based on the location-specific meta-predictors and the RRs, using ‘metan’ package in Stata (version 17.0). The fitted model and the meta-predictors were then used to predict the RRs for each SA2 in Australia.^6,8^ As heat-dominant J-shaped relationship between temperature and health impacts is reported to be more reflective of conditions in most regions of Australia,^9^ we assumed a constant log-linear increase in RRs for each unit increment in temperature exposure above the threshold.^3^ We also asses the non-linear relationships by modifying the modelling functions in our sensitivity analyses.^8^

To determine the counterfactual level of exposure associated with the theoretical minimum risk exposure distributions (TMREDs) for each SA2 in Australia, i.e., the temperature at which the risk of CVD would be lowest, we used the annual most frequent temperatures (MFTs) within the range of the 54^th^ to 92^nd^ percentile, averaged during the baseline period, as our defined TMREDs. Our adoption of this approach was based on previous research conducted in 420 locations globally, which reported comparable values and a consistent association between MFTs and optimal temperature.^10^

Our projections were based on a conservative assumption that the exposure-response relationship (RRs per unit increase in mean temperature) estimated on the current temperature range will remain constant in the future.^11^ In addition, we explored potential human adaptation by shifting the TMREDs under three scenarios: no adaptation, full adaptation, and partial adaptation.^12^ For the ‘no adaptation’ scenario, we used the baseline TMREDs to proceed the calculations, assuming 0% adaptation. For the ‘full adaptation’ scenario, we used TMREDs calculated from future climate data for each projection period, assuming 100% adaptation to higher temperatures. We also considered ‘partial adaptation’ scenario, where people were assumed to adapt to climate change but not fully keep pace with it. In this case, we used the mean of the TMREDs from the ‘no adaptation’ and ‘full adaptation’ scenarios, resulting in a 50% level of adaptation.

**Stage 3: population attributable fraction (PAF) for CVD due to high temperature exposure**

To estimate the PAFs of burden of CVD attributable to high temperature exposure in both the baseline and the future periods, we used the location-specific predicted RRs in combination with observed and projected daily mean temperatures, respectively. The PAFs were calculated for each SA2 per year using the following formula:

$$PAF=\frac{\sum_{c}Pc (RRc-1)}{\sum_{c}Pc \left( RRc-1 \right)+1} \times100$$

*∑_c_* is the sum over all categories, c is an index for 1°C exposure level category, P is the proportion of ‘hot’ days across the year on which temperature was above TMRED, and RR_c_ is the relative risk specific to the temperature category as defined above.

**Stage 4: projection of burden of CVD due to high temperature exposure**

To estimate the burden of CVD attributable to high temperature, we multiplied the YLLs and YLDs with the fatal and non-fatal PAFs, respectively. This provided the annual high temperature attributable burden of CVD in each SA2 area during the baseline (2003-2018) and two future periods (2030s and 2050s). We then estimated the changes in projected burden of CVD associated with high temperatures for the future periods compared to the baseline, under the RCP4.5 and RCP8.5 scenarios. This included the total attributable DALYs, percentage changes, and rates per 100,000 population.

| **RCPs** | **GCMs and Centre** | **Tmean**  **(mean)** | |  | **Tmean (minimum)** | |  | **Tmean (maximum)** | |
| --- | --- | --- | --- | --- | --- | --- | --- | --- | --- |
|  |  | **2030s** | **2050s** |  | **2030s** | **2050s** |  | **2030s** | **2050s** |
| **4.5** | ACCESS1.0 (CSIRO-BOM, Australia) | 18.39 | 18.90 |  | 5.42 | 6.24 |  | 32.12 | 32.58 |
|  | CanESM2 (CCCMA, Canada) | 18.54 | 19.09 |  | 5.62 | 6.12 |  | 32.47 | 33.02 |
|  | CESM1-CAM5 (NSF-DOE-NCAR, USA) | 18.28 | 18.84 |  | 5.60 | 6.02 |  | 31.88 | 32.64 |
|  | CNRM-CM5 (CNRM-CERFACS, France) | 18.17 | 18.59 |  | 5.37 | 5.70 |  | 31.73 | 32.15 |
|  | GFDL-ESM2M (NOAA, GFDL, USA) | 18.39 | 18.60 |  | 5.59 | 5.64 |  | 32.48 | 32.35 |
|  | HadGEM2-CC (MOHC, UK) | 18.41 | 19.12 |  | 5.30 | 6.71 |  | 32.11 | 32.71 |
|  | MIROC5 (JAMSTEC, Japan) | 18.40 | 18.69 |  | 5.55 | 5.62 |  | 31.76 | 32.13 |
|  | NorESM1-M (NCC, Norway) | 18.25 | 18.54 |  | 5.34 | 5.54 |  | 31.75 | 32.07 |
| **8.5** | ACCESS1.0 (CSIRO-BOM, Australia) | 18.52 | 19.28 |  | 5.73 | 6.41 |  | 32.23 | 33.34 |
|  | CanESM2 (CCCMA, Canada) | 18.79 | 19.75 |  | 5.94 | 6.40 |  | 32.67 | 33.83 |
|  | CESM1-CAM5 (NSF-DOE-NCAR, USA) | 18.65 | 19.33 |  | 5.83 | 6.41 |  | 32.16 | 33.32 |
|  | CNRM-CM5 (CNRM-CERFACS, France) | 18.30 | 19.13 |  | 5.28 | 5.97 |  | 31.77 | 32.57 |
|  | GFDL-ESM2M (NOAA, GFDL, USA) | 18.36 | 19.05 |  | 5.47 | 5.85 |  | 32.27 | 33.26 |
|  | HadGEM2-CC (MOHC, UK) | 18.71 | 19.57 |  | 5.88 | 6.86 |  | 32.40 | 33.33 |
|  | MIROC5 (JAMSTEC, Japan) | 18.40 | 19.04 |  | 5.33 | 6.01 |  | 31.91 | 32.65 |
|  | NorESM1-M (NCC, Norway) | 18.40 | 18.97 |  | 5.49 | 6.18 |  | 31.86 | 32.43 |

**Table S1.** Description of global climate models and the projected daily mean temperature (°C) across eight climate models for the 2030s and 2050s (RCP4.5 and RCP8.5).

Source: <https://www.climatechangeinaustralia.gov.au/en/obtain-data/application-ready-data/eight-climate-models-data/>

**
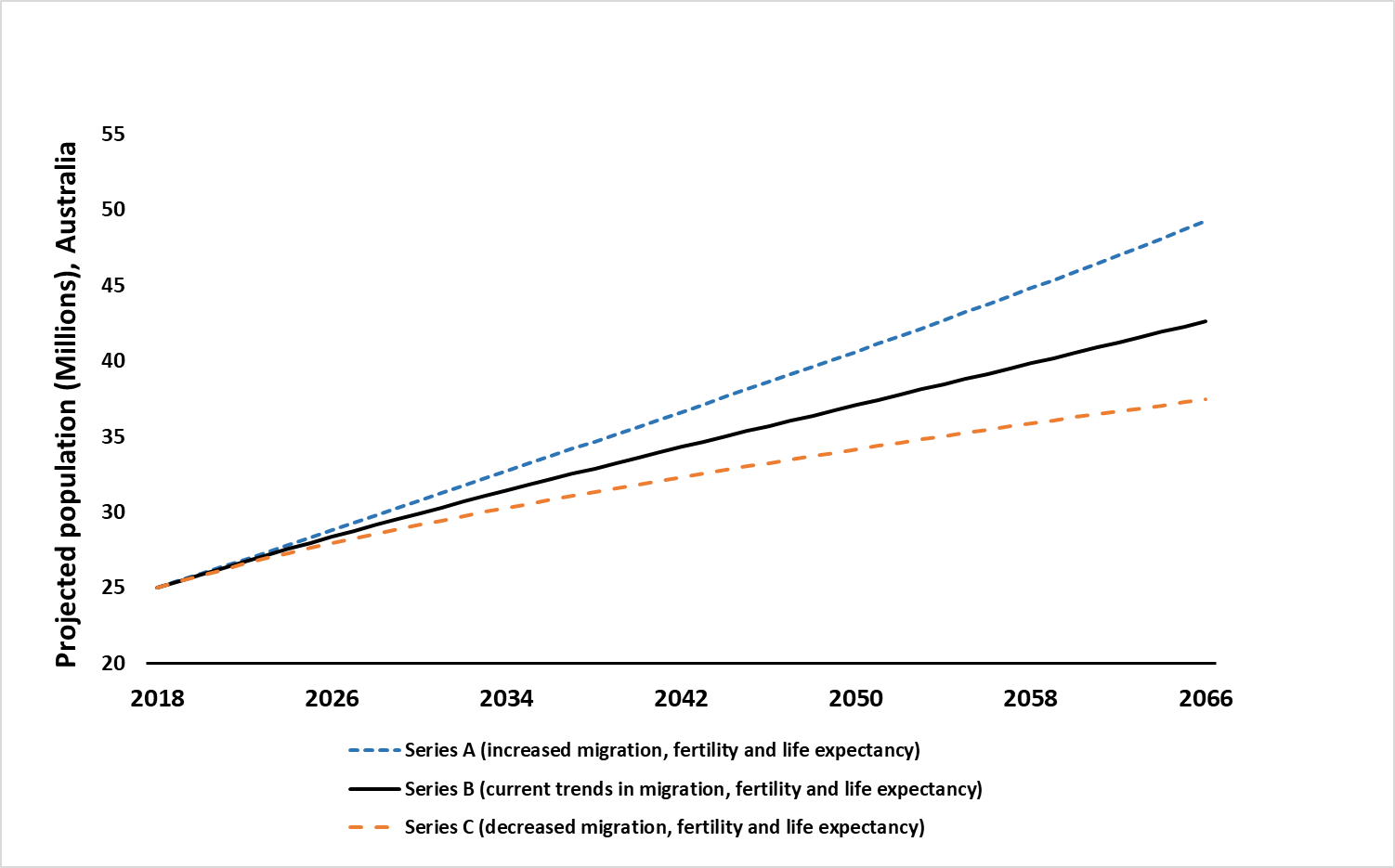
Figure S1.** Total population changes in Australia, by three projection series between 2018 and 2065.

**Table S2.** Burden of cardiovascular disease (CVD) in each state and territory in Australia, 2003-2018.

| **State**  **/Territory** | **Population**  **(SD)** | **Fatal (YLL) burden** | | | **Non-fatal (YLD) burden** | | | **Total (DALY) burden** | | | **% of national DALYs** |
| --- | --- | --- | --- | --- | --- | --- | --- | --- | --- | --- | --- |
|  |  | **Numbers**  **(SD)** | **Rate**  **(SD)** | **Rate**  **ratio** | **Numbers**  **(SD)** | **Rate**  **(SD)** | **Rate**  **ratio** | **Numbers**  **(SD)** | **Rate**  **(SD)** | **Rate**  **ratio** |  |
| **NSW** | 7,203,978  (440,583) | 183,747  (15,335) | 2,309.1  (412.8) | 1.0 | 49,383  (933) | 611.0  (54.5) | 1.0 | 233,130  (14,656) | 2,920.1  (466.6) | 1.0 | 34.3 |
| **Qld** | 4,408,955  (404,557) | 106,726  (7,723) | 2,415.9  (498.0) | 1.1 | 29,101  (2,451) | 643.6  (49.4) | 1.1 | 135,827  (5,843) | 3,059.4  (544.9) | 1.1 | 20.0 |
| **Vic** | 5,554,022  (502,494) | 126,577  (6,198) | 2,107.1  (340.5) | 0.9 | 33,628  (1,673) | 551.6  (41.0) | 0.9 | 160,205  (5,125) | 2,658.7  (381.4) | 0.9 | 23.5 |
| **WA** | 2,305,735  (236,106) | 47,950  (2,025) | 2,129.4  (375.2) | 0.9 | 12,186  (1,009) | 534.7  (41.1) | 0.9 | 60,137  (1,577) | 2,664.1  (414.5) | 0.9 | 8.8 |
| **SA** | 1,630,083  (74,770) | 45,380  (3,697) | 2,301.1  (367.2) | 1.0 | 11,738  (269) | 584.4  (37.6) | 1.0 | 57,118  (3,453) | 2,885.4  (404.6) | 1.0 | 8.4 |
| **Tas** | 505,773  (16,755) | 15,307  (664) | 2,507.3  (307.2) | 1.1 | 2,946  (164) | 476.9  (26.7) | 0.8 | 18,254  (585) | 2,984.2  (332.2) | 1.0 | 2.7 |
| **ACT** | 368,306  (32,480) | 6,045  (336) | 1,919.5  (331.2) | 0.8 | 1,653  (113) | 518.6  (48.1) | 0.9 | 7,698  (363) | 2,438.0  (377.5) | 0.8 | 1.1 |
| **NT** | 227,968  (17,032) | 6,527  (339) | 3,985.0  (786.4) | 1.8 | 1,767  (93) | 1,239.2  (192.0) | 2.1 | 8,294  (270) | 5,224.2  (975.5) | 1.8 | 1.2 |
| **Australia** | **22,204,820**  **(1,717,195)** | **538,260**  **(35,196)** | **2,276.5**  **(401.2)** | **-** | **142,403**  **(6,492)** | **592.2**  **(46.1)** | **-** | **680,663**  **(29,771)** | **2,868.6**  **(446.8)** | **-** | **100.0** |

^1^ Averaged annual total across baseline period, and between-year standard deviation (SD).

^2^ Rates were age-standardized to the 2001 Australian Standard Population and expressed per 100,000 population.

^3^ Rate ratios compare the state/territory rate of burden with the Australian rate of burden.

^4^ Data sources: ABS, and AIHW Australian Burden of Disease Database.

^5^ Abbreviation: NSW, New South Wales. Qld, Queensland. Vic, Victoria. WA, Western Australia. SA, South Australia. Tas, Tasmania. ACT, Australian Capital Territory. NT, North Territory.

**
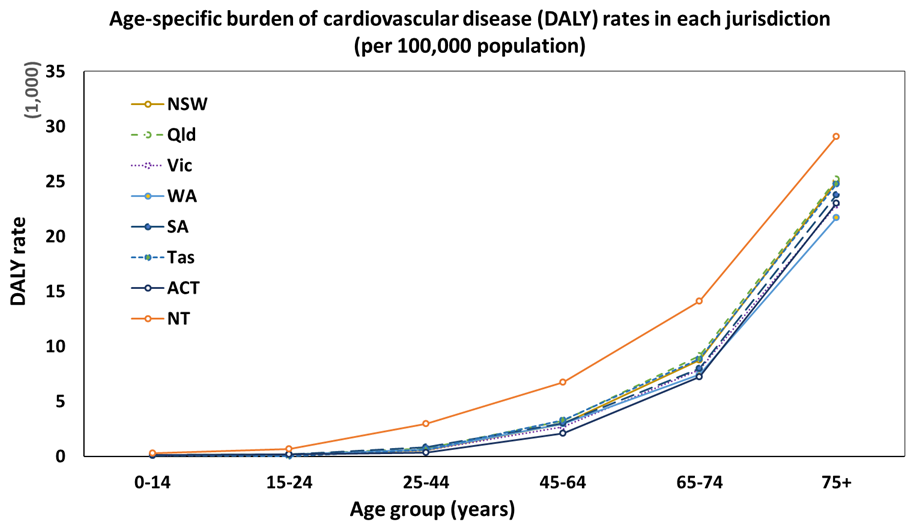
**

**Figure S2.** Age-specific total burden of cardiovascular disease (DALY) rates, by life stage in each jurisdiction (per 100,000 population) during the baseline (2003-2018).


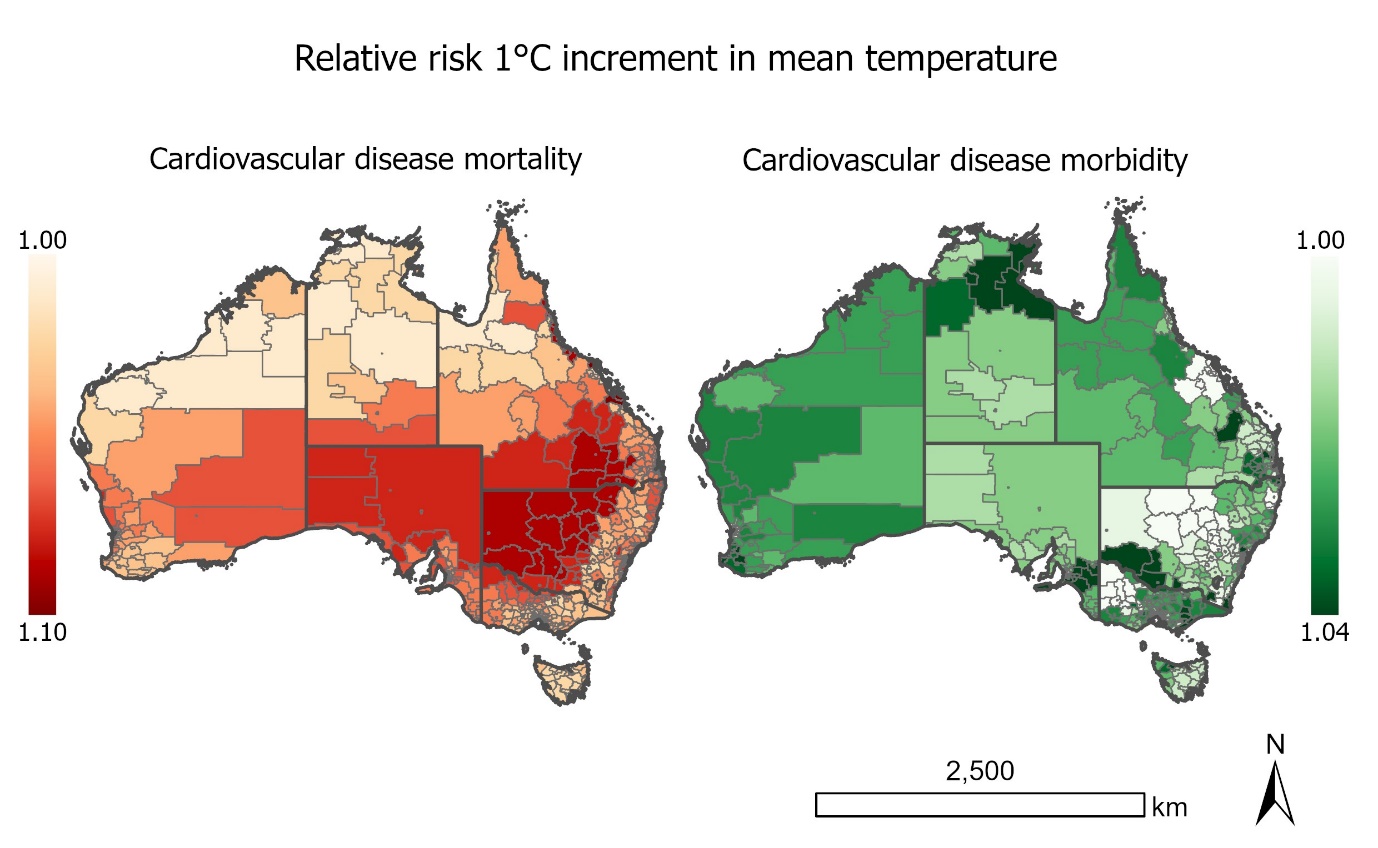


**Figure S3.** Spatial distribution of the relative risk per 1°C increment in mean temperature for cardiovascular disease mortality and morbidity due to high temperatures exposure, by the statistical areas level 2 (SA2) within each state and territory in Australia.

**
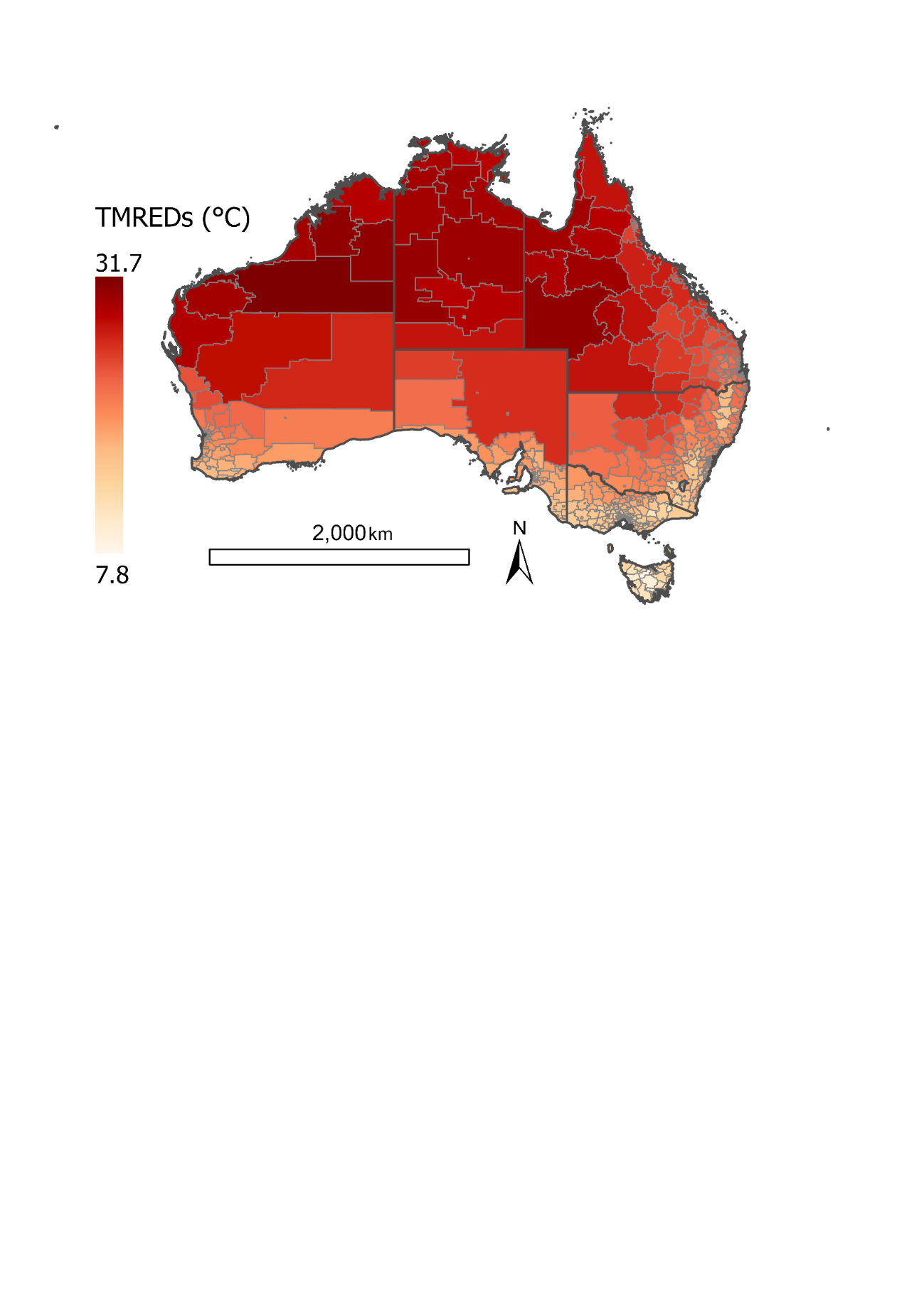
**

**Figure S4.** Spatial distribution of the theoretical minimum risk exposure distributions, TMREDs (°C) in each statistical area level 2 (SA2) within each state and territory in Australia, averaged across the baseline period (2003-2018).

**
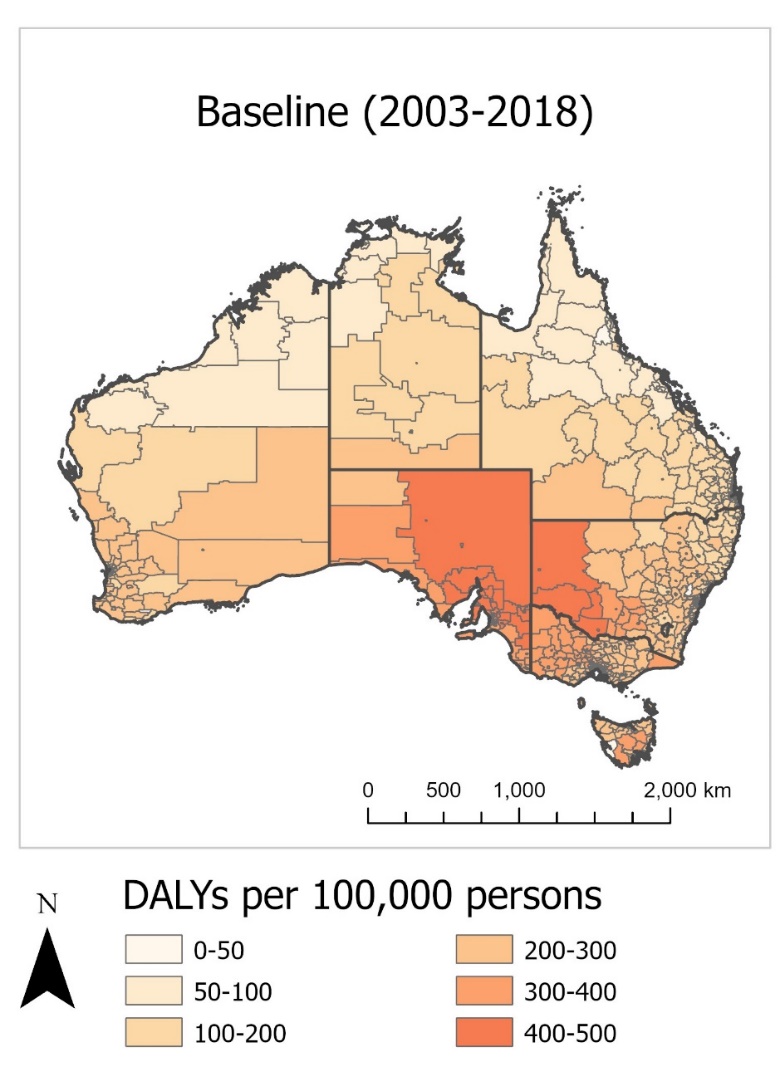
**

**Figure S5.** Estimated annual average rate of the burden of cardiovascular disease attributable to high temperature (per 100,000 population) during the baseline (2003-2018) across Australia.

**Table S3.** Mean (range) in ambient temperature projected by the CMIPS model ensemble, 2016-2045 (referred to as ‘2030s’) and 2036-2065 (referred to as ‘2050s’), under two representative concentration pathways (RCPs), and projected change in population size under different population projection series.

| **Jurisdiction**  **(state and territory)** | **RCPs** | **Projected mean temperature (range) [°C]** | | **Projected change in population size, and percentage**  **[n], and [%]** | | | | | | |
| --- | --- | --- | --- | --- | --- | --- | --- | --- | --- | --- |
|  |  |  |  | **Medium (B) series** | | | **High (A) series** | | **Low (C) series** | |
|  |  | **2030s** | **2050s** | **2030s** | **2050s** | **2030s** | | **2050s** | **2030s** | **2050s** |
| **NSW** | **4.5** | 18.2 (6.4-33.7) | 18.6 (6.9-34.1) | 9,562,802  (132.74%) | 11,845,693  (164.43%) | 9,825,843  (136.39%) | | 12,977,070  (180.14%) | 9,318,128  (129.35%) | 10,911,629  (151.47%) |
|  | **8.5** | 18.3 (6.5-33.8) | 19.2 (7.2-34.7) |  |  |  |  |  |  |  |
| **Qld** | **4.5** | 22.0 (10.3-33.8) | 22.5 (10.8-34.3) | 6,002,898  (136.15%) | 7,435,946  (168.66%) | 6,168,017  (139.9%) | | 8,146,150  (184.76%) | 5,849,307  (132.67%) | 6,849,602  (155.36%) |
|  | **8.5** | 22.2 (10.5-34.0) | 23.0 (11.1-34.8) |  |  |  |  |  |  |  |
| **Vic** | **4.5** | 15.3 (4.4-35.2) | 15.7 (4.8-35.7) | 7,743,573  (139.42%) | 9,592,166  (172.71%) | 7,956,572  (143.26%) | | 10,508,309  (189.20%) | 7,545,445  (135.86%) | 8,835,798  (159.09%) |
|  | **8.5** | 15.4 (4.4-35.4) | 16.1 (5.0-36.1) |  |  |  |  |  |  |  |
| **WA** | **4.5** | 19.4 (8.1-36.2) | 19.8 (8.3-36.6) | 3,108,661  (134.82%) | 3,850,780  (167.01%) | 3,194,170  (138.53%) | | 4,218,566  (182.96%) | 3,029,123  (131.37%) | 3,547,136  (153.84%) |
|  | **8.5** | 19.5 (8.1-36.2) | 20.2 (8.7-37) |  |  |  |  |  |  |  |
| **SA** | **4.5** | 17.2 (6.6-38.1) | 17.6 (6.9-38.4) | 2,080,917  (127.66%) | 2,577,686  (158.13%) | 2,138,156  (131.17%) | | 2,823,879  (173.24%) | 2,027,674  (124.39%) | 2,374,429  (145.66%) |
|  | **8.5** | 17.4 (6.6-38.2) | 18.0 (7.2-38.8) |  |  |  |  |  |  |  |
| **Tas** | **4.5** | 12.5 (1.8-28.8) | 13.0 (2.3-29.3) | 633,071  (125.17%) | 784,201  (155.05) | 650,484  (128.61%) | | 859,100  (169.86%) | 616,873  (121.97%) | 722,365  (142.82%) |
|  | **8.5** | 12.6 (2.0-29.0) | 13.3 (2.6-29.6) |  |  |  |  |  |  |  |
| **ACT** | **4.5** | 14.4 (1.6-31.4) | 14.9 (2.1-31.9) | 503,749  (136.77%) | 624,007  (169.43%) | 517,605  (140.54%) | | 683,606  (185.61%) | 490,860  (133.28%) | 574,802  (156.07%) |
|  | **8.5** | 14.6 (1.7-31.6) | 15.4 (2.3-32.5) |  |  |  |  |  |  |  |
| **NT** | **4.5** | 27.7 (17.1-34.7) | 28.1 (17.5-35.2) | 296,055  (129.87%) | 366,731  (160.87%) | 304,198  (133.44%) | | 401,757  (176.23%) | 288,480  (126.54%) | 337,813  (148.18%) |
|  | **8.5** | 27.9 (17.2-34.8) | 28.6 (17.8-35.6) |  |  |  |  |  |  |  |
| **National** | **4.5** | 18.4 (6.9-34.3) | 18.8 (7.4-34.7) | 29,931,725  (134.80%) | 37,077,210  (166.98%) | 30,755,046  (138.51%) | | 40,618,436  (182.93%) | 29,165,889  (131.35%) | 34,153,575  (153.81%) |
|  | **8.5** | 18.5 (7.0-34.5) | 19.3 (7.7-35.3) |  |  |  |  |  |  |  |

RCP, representative concentration pathways. NSW, New South Wales. Qld, Queensland. Vic, Victoria. WA, Western Australia. SA, South Australia. Tas, Tasmania. ACT, Australian Capital Territory. NT, North Territory. Medium (B) series, current trends in migration, fertility and life expectancy. High (A) series, increased migration, fertility and life expectancy. Low (C) series, decrease migration, fertility and life expectancy.

**
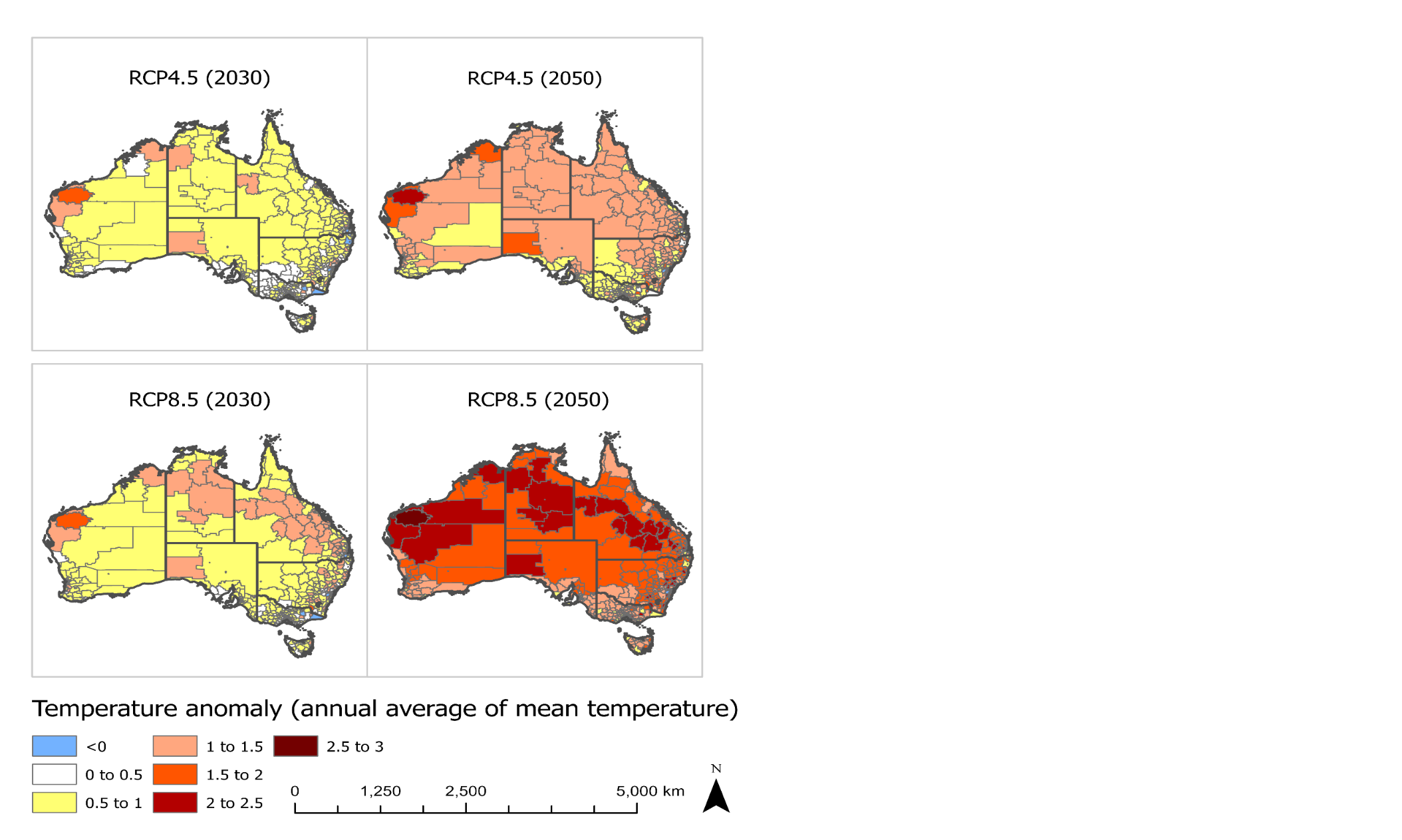
**

**Figure S6.** Projected annual mean temperature (°C) anomaly during 2016-2045 (referred to as ‘2030’), and 2036-2065 (referred to as ‘2050’) verses 2003-2018, under two representative concentration pathways (RCPs).

**Table S4.** Projected changes in proportional of the total annual average population between age groups (0-64 years and over 65 years) for the 2018, 2030 (2026-2035), 2040 (2036-2045), 2050 (2046-2055), and 2060 (2056-2065), by state and territory in Australia.

| **Jurisdictions** | **2018^1^** | | **2030^1^** | | **2040^1^** | | **2050^1^** | | **2060^1^** | |
| --- | --- | --- | --- | --- | --- | --- | --- | --- | --- | --- |
|  | **0-64** | **≥ 65** | **0-64** | **≥ 65** | **0-64** | **≥ 65** | **0-64** | **≥ 65** | **0-64** | **≥ 65** |
| NSW | 83.9% | 16.1% | 81.7% | 18.3% | 81.1% | 18.9% | 80.8% | 19.2% | 79.6% | 20.4% |
| Qld | 84.7% | 15.3% | 81.8% | 18.2% | 80.8% | 19.2% | 80.6% | 19.5% | 79.6% | 20.4% |
| Vic | 84.7% | 15.3% | 82.9% | 17.1% | 82.2% | 17.8% | 81.6% | 18.5% | 80.1% | 19.9% |
| WA | 85.7% | 14.3% | 82.4% | 17.6% | 81.6% | 18.4% | 81.2% | 18.8% | 80.5% | 19.5% |
| SA | 81.6% | 18.4% | 78.0% | 22.1% | 77.1% | 23.0% | 77.2% | 22.8% | 76.7% | 23.3% |
| Tas | 80.3% | 19.7% | 75.7% | 24.3% | 74.5% | 25.5% | 74.6% | 25.4% | 73.6% | 26.4% |
| ACT | 87.3% | 12.7% | 85.3% | 14.7% | 84.6% | 15.4% | 84.1% | 15.9% | 83.1% | 16.9% |
| NT | 92.6% | 7.5% | 90.6% | 9.4% | 90.2% | 9.8% | 89.9% | 10.1% | 89.2% | 10.9% |
| **National** | 84.4% | 15.6% | 81.9% | 18.1% | 81.1% | 18.9% | 80.7% | 19.3% | 79.6% | 20.4% |

^1^Annual average across the time periods

**
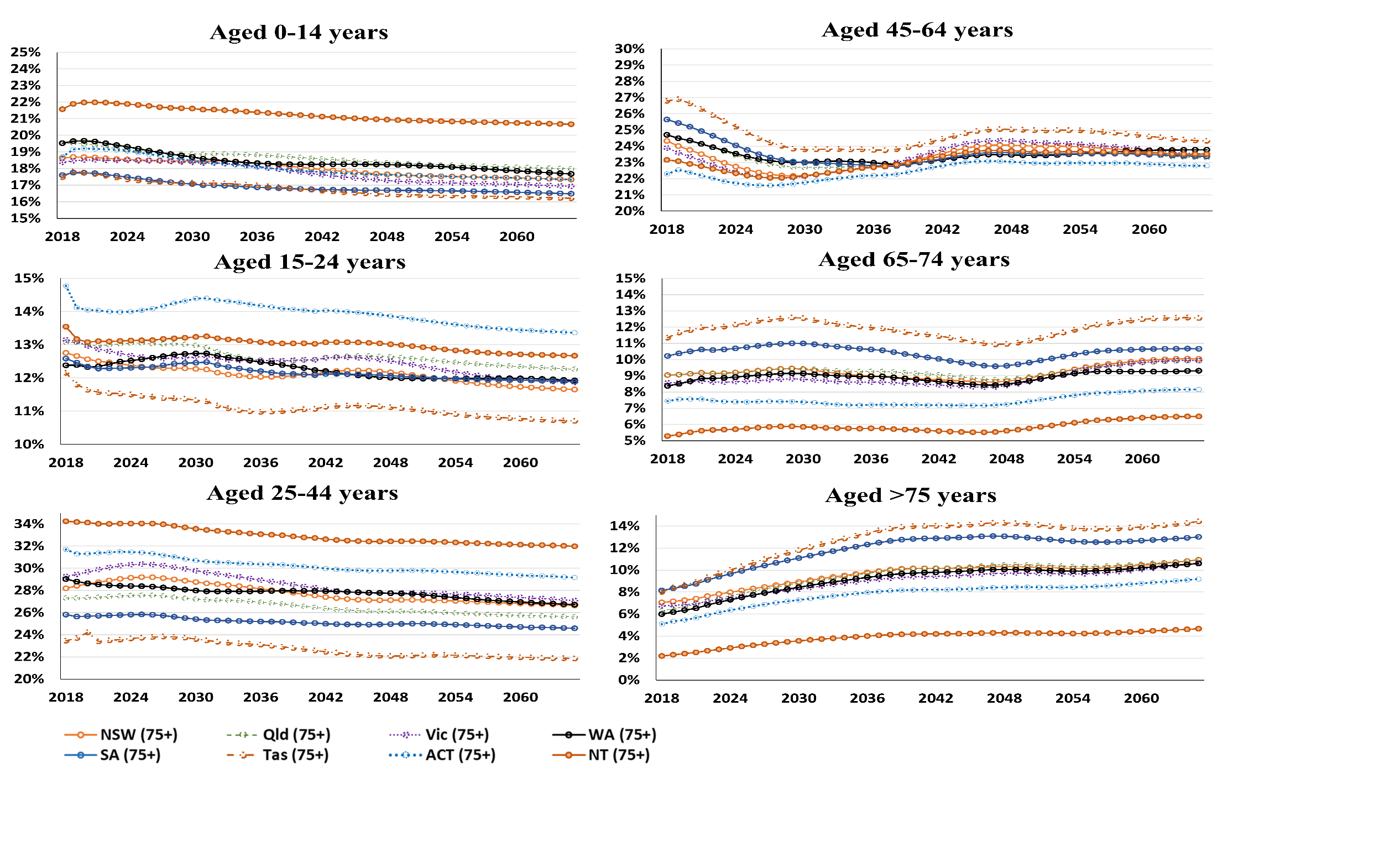
Figure S7.** Projected changes in proportional of population aged 0-14, 15-24, 25-44, 45-64, 65-74, and over 75 years under series B projection, from 2018 to 2065, by state and territory in Australia. NSW, New South Wales. Qld, Queensland. Vic, Victoria. WA, Western Australia. SA, South Australia. Tas, Tasmania. ACT, Australian Capital Territory. NT, North Territory

**
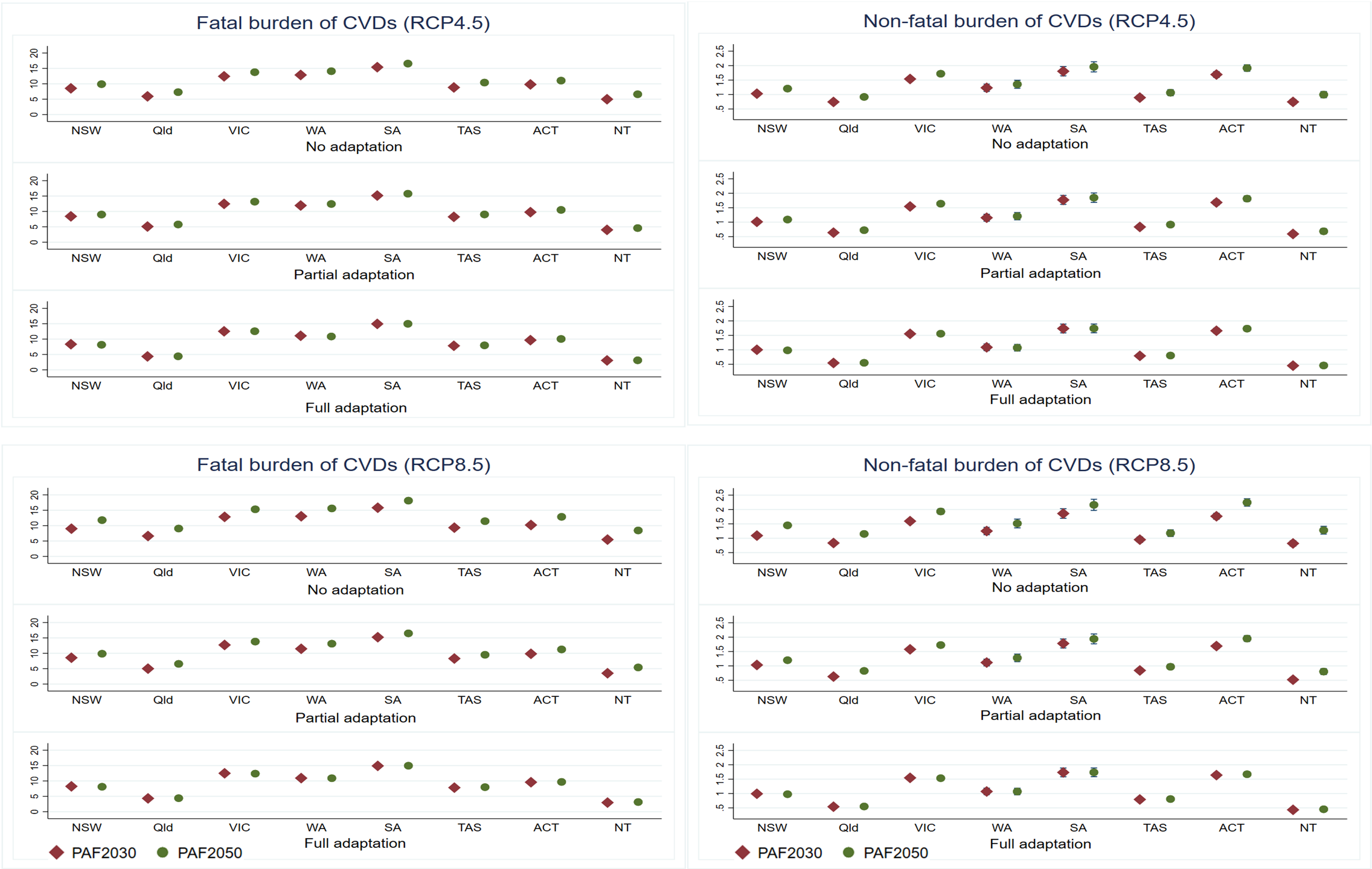
**

**Figure S8.** Projected population attributable fraction for fatal and non-fatal burden of cardiovascular disease due to high temperature exposure for each future period centred on 2030s and 2050s, by state and territory, under two greenhouse gas emission scenarios (RCP4.5 and RCP8.5) and adaptation to climate change (none, partial, full).

**Table S5.** Share of the projected additional burden of cardiovascular diseases (CVDs) for future periods centred on 2030s and 2050s due to different scenarios of climatic, population changes and human adaptation, compared to the baseline of 49483.2 DALYs of the burden of CVDs attributable to high temperature exposure.

| **Time periods** | **Climate scenarios** | **Fraction due to human adaptation** | **Fraction due to changes in temperature (constant population)** | **Fraction due to changes in population** |
| --- | --- | --- | --- | --- |
| **Baseline: 49483.2 DALYs of burden of CVDs attributable to high temperature exposure** | | | | |
| **2030s** | **RCP4.5** | **No** | **+9.9% (4922.9)** | **+73.5% (36373.6)** |
|  |  | **Partial** | +6.5% (3223.9) | +71.0% (35112.7) |
|  |  | **Full** | +3.7% (1813.9) | +68.9% (34085.2) |
|  | **RCP8.5** | **No** | **+15.4% (7639.4)** | **+77.2% (38220.4)** |
|  |  | **Partial** | +7.3% (3597.1) | +71.5 (35355.7) |
|  |  | **Full** | +2.9% (1421.9) | +68.4% (33851.2) |
| **2050s** | **RCP4.5** | **No** | **+25% (12368.0)** | **+157.6% (77977.7)** |
|  |  | **Partial** | +13.7% (6763.0) | +143.0 (70771.7) |
|  |  | **Full** | +3.1% (1521.4) | +129.2% (63945.8) |
|  | **RCP8.5** | **No** | **+44.0% (21765.1)** | **+181.6% (89846.8)** |
|  |  | **Partial** | +22.1% (10930.2) | +153.6% (75994.9) |
|  |  | **Full** | +2.3% (1152.1) | +128.2% (63457.8) |

**Table S6.** Projected proportional of high temperature attributable burden of cardiovascular disease for future periods centred on 2030s, and 2050s (percentage and standard deviation), under scenarios of constant population and medium population growth, adaptation to climate change (none, partial, full), and two representative concentration pathways (RCP4.5, RCP8.5). Estimates are the mean across eight climate models.


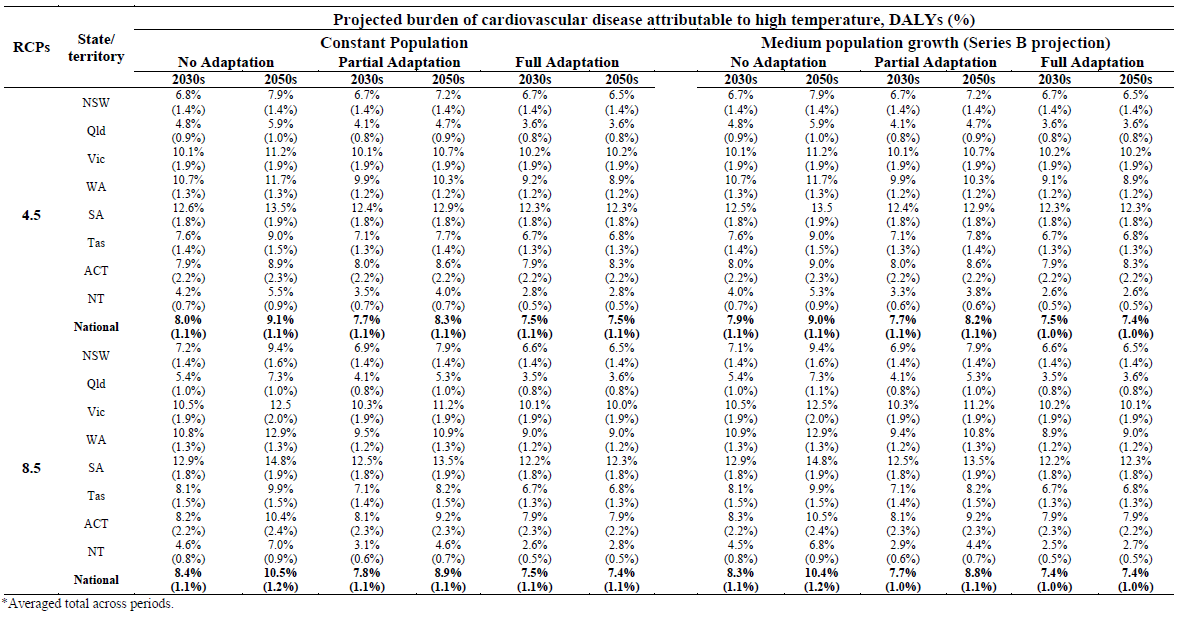


**Table S7.** Projected high temperature-attributable burden of cardiovascular disease (per 100,000 population) for future period centred on 2030s, and 2050s, under scenarios of constant population and medium population growth, adaptation to climate change (none, lagged, on-pace), and two representative concentration pathways (RCP4.5 and RCP8.5). Each estimate gives the mean across eight climate models.


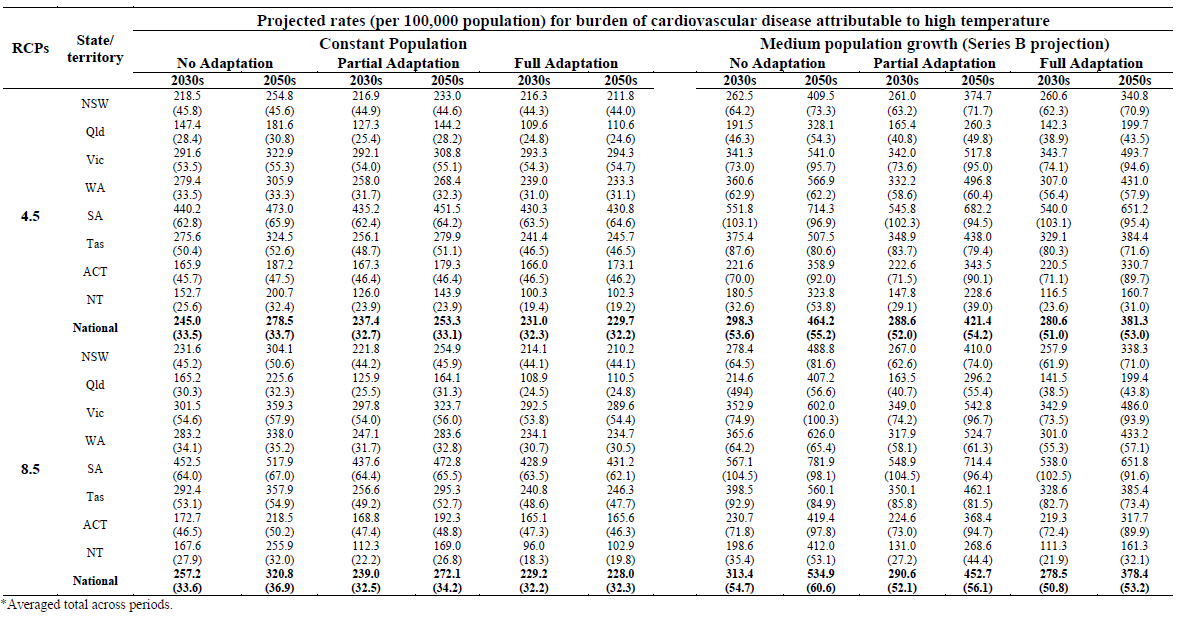


**
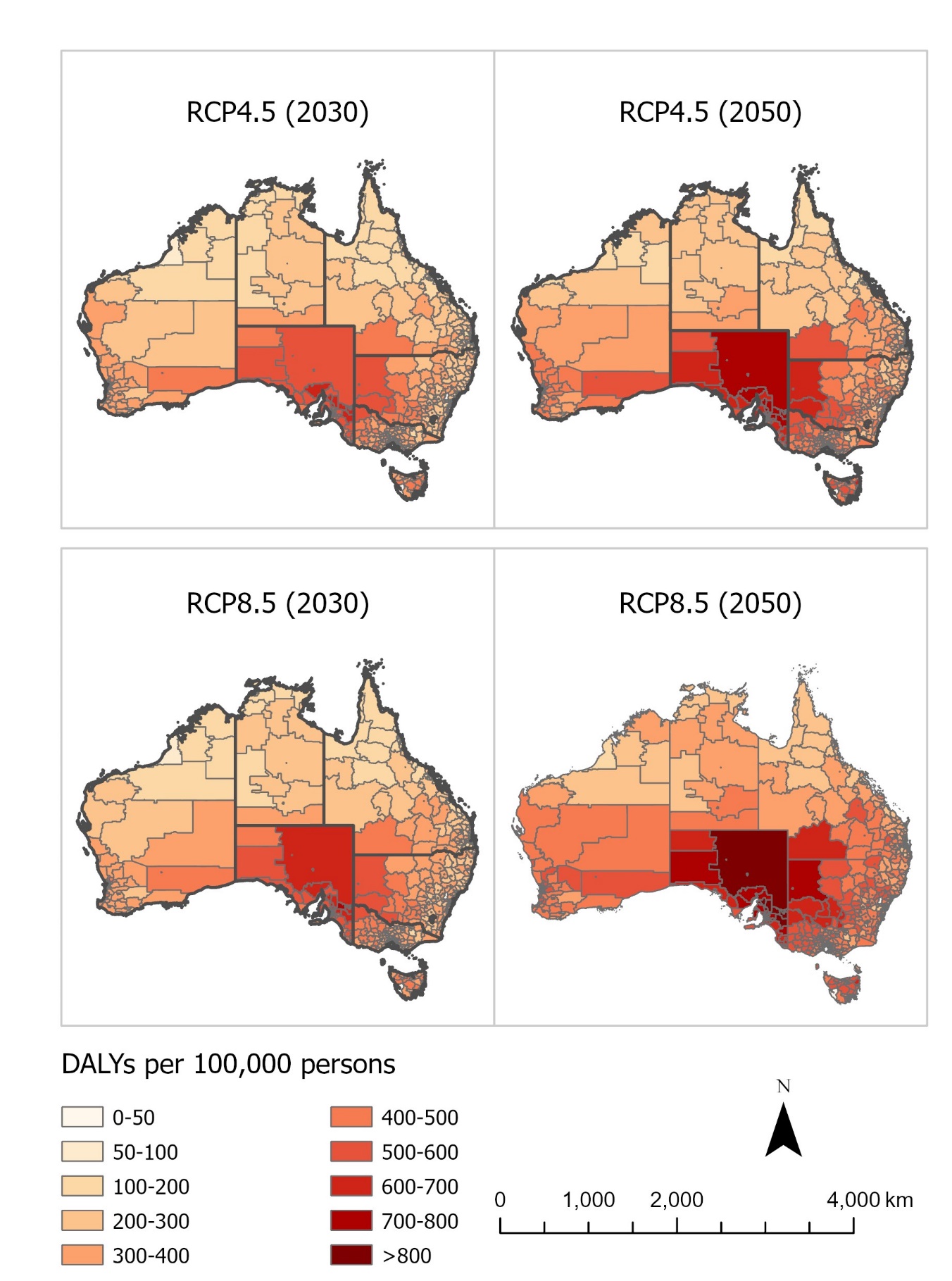
**

**Figure S9.** Projected annual burden of cardiovascular disease attributable to high temperature (per 100,000 population) in each future period centred on 2030s, 2050s **considering no human adaptation** (series B population projection), under two greenhouse gas emission scenarios (RCP4.5 and RCP8.5).

**Table S8.** Sensitivity analysis for the current (2003-2018) heat attributable burden of CVD by varying modelling choices (reference temperature, exposure period, 95%CI of RR, and shape of the exposure-response curve).

| **Modelling choices** | **Heat attributable DALY^1^** | **Heat attributable DALYs,**  **as % of burden of CVD** |
| --- | --- | --- |
| **Reference temperature** |  |  |
| Main model, using most frequent temperature (MFT) within 54^th^ to 92^th^ percentile | | |
| using average mean temperature | 36515 | 5.4% |
| using average median | 38140 | 5.6% |
| **Exposure period** |  |  |
| Main model (using average MFT within 54^th^ to 92^th^ percentile 2003-2018) | | |
| using 2003-2006 average MFT | 54368 | 8.0% |
| using 2007-2010 average MFT | 52546 | 7.7% |
| using 2011-2014 average MFT | 48688 | 7.2% |
| using 2015-2018 average MFT | 44223 | 6.5% |
| **Uncertainty analysis** |  |  |
| Main model (RR from Model I^a^) | | |
| Model II^b^ | 51199 | 7.5% |
| Model III^b^ | 52904 | 7.8% |
| **Shape of the exposure-response curve** |  |  |
| Log-linear (Main model) |  |  |
| Non-linear: quadratic | 53726 | 7.9% |
| Non-linear: cubic | 54058 | 7.9% |

^1^ Average annual total across baseline period, and standard deviation

a Model I, adjusted for annual average temperature, latitude, and climate zone.

b Model II, adjusted for annual average temperature (Tmean), latitude, Gross Domestic Product (GDP) per capita, longitude interaction with continent (Long#cont), continent and climate zone.

**Table S9.** Sensitivity analysis for the future heat attributable burden of CVD by varying modelling choices (reference temperature, projected climate models, and population projection series, shape of the exposure-response curve), **assuming no human adaptation** (series B population projection). Projected disability-adjusted life years (DALYs), and percentage change (%) compared to the baseline.

| **Climate Zones** | **RCP4.5**  **DALYs attributable to high temperatures^1^** | |  | **RCP8.5**  **DALYs attributable to high temperatures^1^** | |
| --- | --- | --- | --- | --- | --- |
|  | **2030s** | **2050s** |  | **2030s** | **2050s** |
| **Reference temperature** | | | | | |
| Main model (using MFT within 54^th^ to 92^th^ percentile) | | | | | |
| using average mean temperature | 68109.2 (37.6%) | 106737.8 (115.7%) |  | 71997.6 (45.5%) | 125391.8 (153.4%) |
| using average median | 70898.6 (43.3%) | 110746.7 (123.8%) |  | 74763.9 (51.1%) | 129941.2 (162.6%) |
| **Projected climate models** | | | | | |
| Main model (averaged data from all the eight models) | | | | | |
| ACCESS1.0 | 92422.6 (86.8%) | 157474.1 (218.2%) |  | 94415.1 (90.8%) | 162662.5 (228.7%) |
| CanESM2 | 98394.7 (98.8%) | 157977.3 (219.3%) |  | 106652.6 (115.5%) | 189364.9 (282.7%) |
| CESM1-CAM5 | 87294.8 (76.4%) | 138122.2 (179.1%) |  | 98702.6 (99.5%) | 162000.4 (227.4%) |
| CNRM-CM5 | 87030.0 (75.9%) | 132767.7 (168.3%) |  | 92000.0 (85.9%) | 160819.8 (225.0%) |
| GFDL-ESM2M | 91969.6 (85.9%) | 129405.3 (161.5%) |  | 88909.8 (79.7%) | 152002.7 (207.2%) |
| HadGEM2-CC | 91887.5 (85.7%) | 153971.0 (211.2%) |  | 99548.0 (101.2%) | 172372.1 (248.3%) |
| MIROC | 91685.4 (85.3%) | 135741.8 (174.3%) |  | 93638.5 (89.2%) | 148771.0 (200.6%) |
| NorESM1-M | 87492.0 (76.8%) | 132242.6 (167.2%) |  | 91011.6 (83.9%) | 145204.5 (193.4%) |
| **Population projection series** | | | | | |
| Main model (series B-current trends in migration, fertility and life expectancy) | | | | | |
| Series A (increase migration, fertility and life expectancy) | 92931.5 (87.8%) | 152658.7 (208.5%) |  | 97607.0 (97.3%) | 175870.0 (255.4%) |
| Series C (decrease migration, fertility and life expectancy) | 90284.4 (82.5%) | 137023.9 (176.9%) |  | 94239.1 (90.4%) | 157884.6 (219.1%) |
| **Shape of the exposure-response curve** | | | | | |
| Main model (Log-linear) | | | | | |
| Non-linear: quadratic | 98469.5 (99.0%) | 151546.6 (206.3%) |  | 103386.1 (108.9%) | 174427.2 (252.5%) |
| Non-linear: cubic | 99070.4 (100.2%) | 152460.8 (208.1%) |  | 104014.4 (110.2%) | 175466.1 (254.6%) |

^1^Average across the projected scenario periods centred on the 2030 (2016-2045), and 2050 (2036-2065).

**References**

1. Gasparrini A, Masselot P, Scortichini M, et al. Small-area assessment of temperature-related mortality risks in England and Wales: a case time series analysis. Lancet Planet Heal. 2022;6(7):e557-e564. doi:10.1016/S2542-5196(22)00138-3

2. Yang J, Zhou M, Ren Z, et al. Projecting heat-related excess mortality under climate change scenarios in China. Nat Commun. 2021;12(1). doi:10.1038/s41467-021-21305-1

3. Liu J, Varghese BM, Hansen A, et al. Heat exposure and cardiovascular health outcomes: a systematic review and meta-analysis. Lancet Planet Heal. 2022;6(6):e484-e495. doi:10.1016/S2542-5196(22)00117-6

4. Chung SE, Cheong HK, Park JH, Kim JH, Han H. Current and Projected Burden of Disease From High Ambient Temperature in Korea. Epidemiology. 2017;28 Suppl 1:S98-S105. doi:10.1097/EDE.0000000000000731

5. Beck HE, Zimmermann NE, McVicar TR, Vergopolan N, Berg A, Wood EF. Present and future Köppen-Geiger climate classification maps at 1-km resolution. Sci Data. 2018;5(1):180214. doi:10.1038/sdata.2018.214

6. Zhao Q, Guo Y, Ye T, et al. Global, regional, and national burden of mortality associated with non-optimal ambient temperatures from 2000 to 2019: a three-stage modelling study. Lancet Planet Heal. 2021;5(7):e415-e425. doi:10.1016/S2542-5196(21)00081-4

7. Gasparrini A, Guo Y, Sera F, et al. Projections of temperature-related excess mortality under climate change scenarios. Lancet Planet Heal. 2017;1(9):e360-e367. doi:10.1016/S2542-5196(17)30156-0

8. Liu J, Hansen A, Varghese BM, et al. Estimating the burden of disease attributable to high ambient temperature across climate zones: methodological framework with a case study. Int J Epidemiol. Published online 2022:1-13. doi:10.1093/ije/dyac229

9. Longden T. The impact of temperature on mortality across different climate zones. Clim Change. 2019;157(2):221-242. doi:10.1007/s10584-019-02519-1

10. Yin Q, Wang J, Ren Z, Li J, Guo Y. Mapping the increased minimum mortality temperatures in the context of global climate change. Nat Commun. 2019;10(1):4640. doi:10.1038/s41467-019-12663-y

11. Vicedo-Cabrera AM, Sera F, Gasparrini A. Hands-on Tutorial on a Modeling Framework for Projections of Climate Change Impacts on Health. Epidemiology. 2019;30(3):321-329. doi:10.1097/EDE.0000000000000982

12. Anderson GB, Oleson KW, Jones B, Peng RD. Projected trends in high-mortality heatwaves under different scenarios of climate, population, and adaptation in 82 US communities. Clim Change. 2018;146(3-4):455-470. doi:10.1007/s10584-016-1779-x
